# Measurement and Modeling of Ship-Related Ultrafine Particles and Secondary Organic Aerosols in a Mediterranean Port City

**DOI:** 10.3390/toxics11090771

**Published:** 2023-09-11

**Authors:** Matthias Karl, Martin Otto Paul Ramacher, Sonia Oppo, Ludovic Lanzi, Elisa Majamäki, Jukka-Pekka Jalkanen, Grazia Maria Lanzafame, Brice Temime-Roussel, Lise Le Berre, Barbara D’Anna

**Affiliations:** 1 Department of Coastal Environmental Chemistry, Helmholtz Zentrum Hereon, 21502 Geesthacht, Germany; martin.ramacher@hereon.de; 2 AtmoSud, Air Quality Observatory in the Provence-Alpes-Côte d’Azur Region, 13006 Marseille, France; sonia.oppo@atmosud.org (S.O.); ludovic.lanzi@atmosud.org (L.L.); 3 FMI (Finnish Meteorological Institute), 00560 Helsinki, Finland; elisa.majamaki@fmi.fi (E.M.); jukka-pekka.jalkanen@fmi.fi (J.-P.J.); 4 CNRS, Laboratoire de Chimie de l’Environnement (LCE), Aix Marseille Université, 13003 Marseille, France; grazia-maria.lanzafame@univ-amu.fr (G.M.L.); brice.temime-roussel@univ-amu.fr (B.T.-R.); lise.le-berre@etu.univ-amu.fr (L.L.B.); barbara.danna@univ-amu.fr (B.D.)

**Keywords:** ultrafine particles, secondary organic aerosols, urban air quality, ship emissions, chemistry transport model, particle number size distribution

## Abstract

Maritime transport emerges as a major source of ultrafine particle (UFP) pollution in coastal regions with consequences for the health of people living in port cities. Inhalation of UFPs can cause inflammation and oxidative stress, which are starting points for further diseases. In addition to primary particles, secondary organic aerosol (SOA) may form through the photo-oxidation of volatile organic compounds emitted in ship exhaust. The characterization of size-segregated and chemical properties of particles is essential for assessing the health implications related to shipping. We applied a coupled regional–local chemistry transport modeling system to study the effects of ship emissions on atmospheric concentrations of UFP and SOA in the Mediterranean port city Marseille (France), which is characterized by the combination of high port activity, industrialized emissions, and active photochemistry in summer. Our results show that the average potential impact from local shipping in the port area was 6–9% for SOA and 27–51% for total particle number concentration in July 2020. The estimated oxidative potential of daily mean particulate organic matter related to shipping was lower than the oxidative potential reported for heavy fuel oil (HFO). The lower oxidative potential in this study is very likely due to the low share of ships using HFO during stopover.

## 1. Introduction

Exhaust particles emitted from oceangoing ships pose a health risk to the population living in port cities and coastal areas with dense ship traffic [1]. In coastal Mediterranean cities, shipping activities are an important contributor to emissions of fine particulate matter with aerodynamic diameters of less than 10 µm (PM_10_) and less than 2.5 µm (PM_2.5_) within the urban area [2,3,4,5]. Furthermore, ship emissions generated while the vessels are at berth docked and maneuvering in the port can have a substantial negative effect on local air quality [6,7]. The amounts and the size spectrum of emitted fine particulate matter depend on ship engine type, engine operation, fuel type, and sulfur content in the fuel [8]. The worldwide introduction of low-sulfur ship fuels has been shown to reduce ship-related mortality and morbidity by 34% and 54%, respectively, in nations across the globe [9].

Shipping emerges as a major source of ultrafine particle (UFP) pollution in coastal cities [7,10]. UFPs typically account for 80–90% of the total particle number (PN) concentration in the urban atmosphere [11]. Ultrafine particles are the fraction of the particle population with an aerodynamic diameter below 100 nm (i.e., 0.1 µm), associated with a large surface area. Particle surface area, the number of UFP, the presence of bioavailable transition metals, polycyclic aromatic hydrocarbons (PAH), and other particle-bound organic compounds appear to be more important than particle mass in determining adverse effects of combustion particles on human health [12]. In this regard, it is notable that measurements in the port of Venice revealed a direct contribution of ship traffic to PAH concentration in the gas phase of 10% [3]. However, more studies are needed on the toxicological effects of these constituents of diesel exhaust, their combined effect, and how they relate to fuel quality and particle chemical characteristics [13].

Inhalation of UFP is associated with inflammation and oxidative stress [13]. Due to their small size, UFP can penetrate deeply into the lungs, from where they can be distributed to other organs of the human body and subsequently trigger biological responses, such as cardiovascular diseases and pulmonary inflammation, and induce nervous disorders due to the attached transition metals, PAHs and organic compounds [13,14]. Oxidative stress occurs when concentrations of reactive oxygen species (ROS) within human lung cells overwhelm cellular anti-oxidant defenses. The capacity of particles to produce ROS with subsequent depletion of anti-oxidants is defined as oxidative potential (OP). OP is considered a relevant metric for the acute health effects of particulate matter upon inhalation [15]. A systematic review of epidemiological studies on the health effects of UFP suggests increasing evidence for short-term health effects independent of particulate matter, whereas the evidence is still inconsistent to draw firm conclusions on long-term effects of UFP, mortality, and morbidity [16].

The overwhelming majority of particles emitted in fresh ship exhaust is in the ultrafine size range [7,17]. Therefore, number-based concentrations of particles could be a better metric for determining impacts from shipping, compared to particle mass concentrations [18]. However, only a few studies investigated the size-resolved contribution of shipping to particles in terms of number size distribution (PNSD). Direct measurements of sub-micrometer particles in ship plumes in marine ports revealed a bimodal number size distribution with dominant modes peaking at 20–40 nm and 70–90 nm, with strongly increased numbers compared to the background air [19,20]. Measurements in individual ship plumes at the entrance of the port of Gothenburg, Sweden, revealed that on average 36–46% of the ship-emitted particles by number were non-volatile [21]. Urban air quality modeling showed the influence of shipping on UFP concentrations in major ports [22].

Ship exhaust plumes contain primary, delayed primary, and secondary particles. Primary particles in ship exhaust are emitted during expansion stroke (4-stroke engines) and power stroke (2-stroke engines) because of incomplete combustion of hydrocarbons in the fuel. They consist of soot spherules, which increase to sizes of 10–100 nm through aggregation and coagulation in the form of aggregates and chains [23,24] on a short timescale in the presence of water and sulfuric acid (H_2_SO_4_). In addition to primary particles, volatile organic gases in the emission may undergo gas-to-particle transformation during exhaust dilution and cooling to form volatile particles, referred to as delayed primary particles, usually in a distinct mode of smaller than 25 nm in diameter [22,25]. A possible source of semi-volatile hydrocarbons is the evaporation of lubricating oils from the cylinder liner during the expansion stroke, dominated by polycycloalkanes in the C_20_-to-C_39_ range [26].

Secondary particles form in the ship exhaust by atmospheric oxidation leading to increases in the particle mass of organics, ammonium, nitrate, and sulfate. Secondary organic aerosol (SOA) may form in ship plumes through the chemical oxidation of ship-emitted volatile organic compounds (VOC) by ozone (O_3_) and atmospheric radicals. VOC emissions from auxiliary engines of container ships at berth are dominated by alkanes and aromatics [27]. The composition of VOC emissions from ships may largely vary with the used ship fuel [28]. Airborne measurements in ship plumes of a cruising vessel revealed that the mass ratio of particulate organic carbon (OC) to sulfate between the ship stack and the airborne plume increased from 0.23 to 0.30, with the additional organic mass mainly below 100 nm diameter [29]. The organic-to-sulfate mass ratio remained constant during the first hour of plume dilution in the marine boundary layer, indicating that the formation of SOA takes place in proximity to the ship.

Knowledge gaps regarding the size-segregated and chemical characterization of ship-related particles currently impede the source apportionment of ambient particles in coastal cities and need to be addressed for a better assessment of health implications related to shipping [5]. While a large number of studies investigated the effect of ship-related PM_2.5_ in coastal areas, currently only a few studies [7,10,22,30] deal with the effect of shipping on the number concentrations of UFP and its chemical composition in port cities.

In this study, we employ a coupled regional-to-local chemistry transport model (CTM) system to investigate the effect of ship emissions on SOA mass concentrations and number concentrations of UFP in the port city of Marseille in southern France, which is an important hub of ferry and cruise ship traffic in the Mediterranean Sea. Marseille is the second most populated city in France (870,000 inhabitants and more than 1.5 million in the urban district) and it is the most important port city in the country. This work aims to identify the impacts of ship exhaust particles on UFP pollution and SOA mass concentrations in Marseille during July 2020 and specifically analyzes the number size distribution, concentrations of individual VOCs that act as precursors to SOA formation, and the chemical composition of SOA. We reconstruct the mass concentrations of organic aerosol related to shipping and connect the particulate organic matter (POM) to estimates of oxidative potential to assess the potential health implications of shipping in the port area. Finally, we discuss uncertainties associated with the simulated formation of ship-related SOA with respect to the temperature dependence of gas-particle partitioning of semi-volatile organics, availability of particle surfaces, and oxidative aging during atmospheric transport.

## 2. Materials and Methods

For city-scale simulations of Marseille, EPISODE-CityChem [31] was applied in a coupled setup with the regional-scale Community Multiscale Air Quality Modeling System (CMAQ; [32]). Simulations with the coupled setup were performed for the months of June–October 2020. The model run with EPISODE-CityChem for July 2020 considered the formation of SOA in the urban area and was used in this study for the analysis of modeled UFP and SOA concentrations in Marseille. The first objective has been to investigate the potential impact of shipping in the coastal area and the port of Marseille on urban concentrations of ultrafine particles and SOA mass. A second objective has been to develop a method for the detection of ship exhaust plumes using measured and modeled total PN concentrations. Hourly model output of EPISODE-CityChem for July 2020 was compared to measurements at monitoring stations in Marseille operated by AtmoSud and campaign data recorded at the port site. A third objective has been to estimate the oxidative potential of ship-related POM based on air quality modeling and reported OP parameters from the literature.

### 2.1. Coupled Regional–Local Chemistry Transport Modeling

#### 2.1.1. Model System

**Figure 1:**
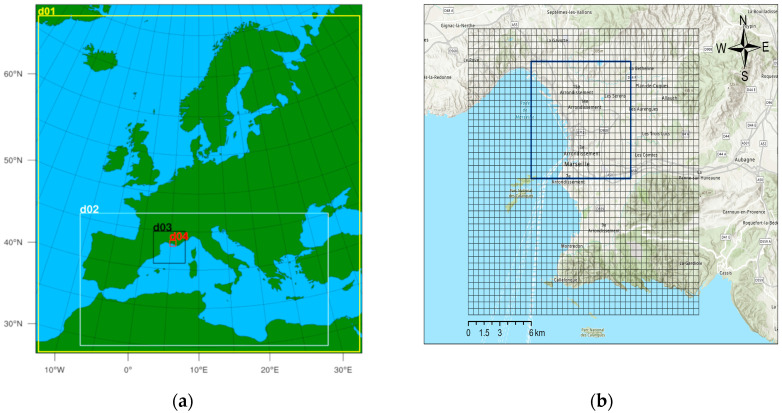
Maps of computational domains of the coupled CTM setup for Marseille simulations: (**a**) nested domains for CMAQ simulations over Europe with horizontal grid resolutions 36 km × 36 km (yellow frame, d01), 12 km × 12 km (white frame, d02), and 4 km × 4 km (black frame, d03); (**b**) urban domain for EPISODE-CityChem simulations with extent of 22 km × 22 km and grid resolution of 500 m × 500 m (zoom into area indicated by red frame, d04, in figure part (**a**). Dark blue frame in figure part (**b**) indicates the extent of the port city area.

The city-scale urban air quality model EPISODE-CityChem used the time-varying three-dimensional (3D) concentration field from CMAQ at the lateral and vertical boundaries as initial and boundary concentrations for selected chemical species. The boundary conditions of the urban domain of Marseille were based on results from regional CMAQ simulations over Europe with finer grid resolution nests over the Mediterranean Sea and Southern France. The three computational domains of the regional scale simulations and the model domain of the city scale simulation for Marseille are shown in Figure 1.

During summertime, Marseille provides ideal conditions for this investigation because of the combination of high shipping activity, urban industrialized emissions, and active photochemistry with high O_3_ concentrations. The coastal environment of Marseille is characterized by a complex topography that leads to two specific regional wind patterns in summer: the diurnal sea/land breeze cycle and the mistral winds [33]. The mistral wind originates from accelerated winds from the north or northwest. Once this strong wind reaches Marseille it is slowed down due to the rough surfaces of the urban terrain. During the daytime, the sea breeze carries industrial emissions from the coastal area (Fos-Berre) that lies to the northwest of Marseille, which may induce the formation of secondary pollutants [34]. At nighttime, the land breeze develops and brings aged air masses from eastern directions to Marseille.

**Figure 2:**
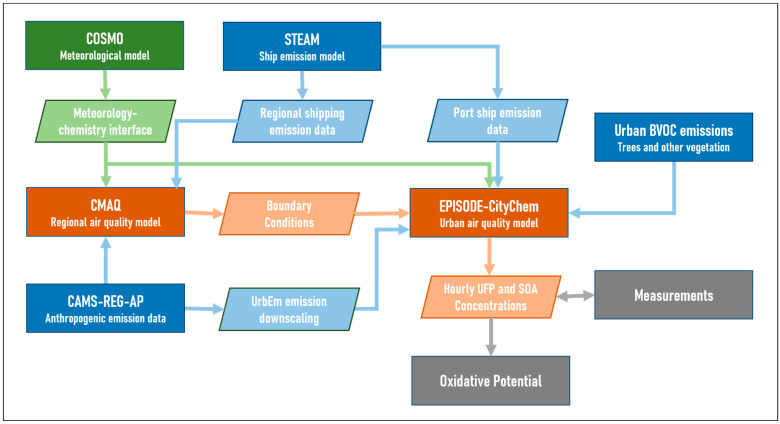
Workflow of the coupled regional-to-local model system. Schemes follow the same formatting. Outline of the sequential processing chain developed in this study. Meteorological components in green colors, emission components in blue colors, and chemistry transport modeling components in brown colors. Comparison to measurements and estimation of oxidative potential are presented in grey colors.

Figure 2 outlines the workflow of the coupled model system used in this study. The city-scale CTM was one-way coupled with the regional-scale CTM through the chemical boundary conditions. To achieve high consistency between the coupled simulations, the same model or data basis for creating anthropogenic emissions has been used in the regional and urban scale simulations. High-resolution meteorological data obtained from the meteorological model COSMO [35] on a 0.022° × 0.022° grid resolution was used for the CMAQ simulation of the 4 km nest and the city-scale simulation with EPISODE-CityChem. The hourly model output of UFP and VOC concentrations was compared to measurements of the field campaign in July 2020. The spatial averages of modeled concentrations within the port city area (frame in Figure 1b) attributable to ship emissions were used to estimate the OP of ship-related UFP and POM.

#### 2.1.2. Regional-Scale CTM Simulation

The CMAQ model v5.2 with the aero6 model [32,36,37] computes the air concentration and deposition fluxes of atmospheric gases and aerosols as a consequence of emission, transport, and chemical transformation. Regional-scale simulations with CMAQ were performed for the months of June to October 2020 for the entirety of Europe (grid cell size: 36 km × 36 km) with nested grids for the Mediterranean Sea (12 km × 12 km) and Southern France (4 km × 4 km).

Fink et al. [38], using CMAQ in the same setup for the simulation of air quality and ship impact in the Mediterranean Sea for the year 2015 evaluated the performance of CMAQ and other regional-scale CTMs against measurement data of the European Environment Agency’s (EEA) monitoring network. For CMAQ, a fair correlation (r = 0.42) for nitrogen dioxide (NO_2_) and a good correlation for O_3_ (r = 0.60) was found when comparing modeled and measured daily mean concentrations. All CTMs included in the intercomparison underestimated the annual mean concentrations of NO_2_ at most measurement sites.

The atmospheric chemistry in CMAQ is treated using the Carbon Bond 05 mechanism [39] with updated toluene chemistry [40] including the chlorine chemistry extension (CB05-TUCL). The gas phase–aerosol partition equilibrium of secondary inorganic aerosols is solved by the ISORROPIA mechanism [41]. The aero6 mechanism in CMAQ includes secondary organic aerosol (SOA) formation from isoprene, terpenes, benzene, toluene, xylene, and alkanes [42].

CMAQ was set up with 30 vertical layers reaching approx. 15 km altitude, with the lowest layer from 0 m to 42 m height. The driving data for chemical boundary conditions was IFS-CAMS cycle45r1 (Integrated Forecasting System—Copernicus Atmosphere Monitoring Service; [43]) with a vertical resolution of 60 sigma levels up to 65 km.

#### 2.1.3. City-Scale CTM

The city-scale model EPISODE-CityChem v1.7 [31,44,45] combines a 3D Eulerian grid model with a sub-grid Gaussian dispersion model to resolve pollutant dispersion in the proximity of point sources and line sources. The Eulerian grid model computes averaged air concentrations of chemical species by solving the advection–diffusion and mass conservation equations. The 3D Eulerian grid model solves the photochemistry of multiple reactive pollutants and includes the various chemical interactions involving nitrogen oxides, ozone, VOC, sulfur dioxide (SO_2_) and other secondary pollutants relevant to the urban atmosphere. The chemistry mechanism EmChem09-HET was applied which contains 80 chemical species, including 12 different VOC, and comprises a total of 106 reactions. The chemistry scheme considers the gas-phase oxidation of individual hydrocarbons (surrogates) by the hydroxyl radical (OH), the nitrate radical (NO_3_), and ozone.

EPISODE-CityChem includes a simplified street canyon model (SSCM) for the calculation of near-source dispersion of pollutant emissions from inner-city vehicular traffic. SSCM is based on the parameterizations of the Operational Street Pollution Model (OSPM; [46]). Three generic types of street canyon geometries are applied, depending on the urban land use classification. The compact chemical reaction scheme EP10-Plume is applied in connection with SSCM, which considers the fast reactions of nitrogen oxides and the photochemical degradation of formaldehyde, an important constituent of vehicle exhaust.

The P8P + 2 parametrization scheme for particle number concentration and PNSD calculation [30] calculates dry deposition and coagulation (between particles of the same size class) of each size class of particles. Ten particle size classes are defined in the P8P + 2 scheme, covering the particle diameter size range between 0.001 µm and 10 µm (Table S1, Supplementary Materials). The wet scavenging of particles is parameterized based on the formulation by Sič et al. [47] when considering in-cloud scavenging and below-cloud scavenging of particles with different sizes. The P8P + 2 scheme is implemented in the Eulerian grid model and the (sub-grid) Gaussian models. The accuracy of the implemented parameterization for aerosol processes for the prediction of PN concentrations is limited by three factors: first, by the averaging of process parameters over a certain size range; second, by the simplified treatment of coagulation; and third, by neglecting condensation and evaporation. Compared to a fully size-resolved aerosol dynamics model, calculated total particle number concentrations have an error of approximately 10% [48].

Concentrations of particulate matter (PM_2.5_ and PM_10_) in the model are controlled by their primary emissions (from point, line, and area sources), the intrusion through the domain boundaries, and the atmospheric dispersion. Dry and wet deposition processes for gases and particulate matter are also included. The implementation of SOA formation in EPISODE-CityChem is described in the following subsection.

#### 2.1.4. City-Scale SOA Modeling

For solving SOA formation in the 3D Eulerian grid model, the condensation/evaporation and the SOA module from MAFOR v.2 [49] were incorporated in EPISODE-CityChem v1.7 and coupled to the P8P + 2 scheme. The functional structure of the improved EPISODE-CityChem model with the coupling of SOA formation to the particle number scheme is illustrated in Figure S1, Supplementary Materials. The Analytical Predictor of Condensation scheme [50] is employed in the condensation/evaporation module to calculate the mass transfer of gas molecules to particles. The Analytical Predictor of Condensation, with applied mass balance restrictions, is unconditionally stable. The SOA formation module relies on the 2D Volatility Base Set (VBS) framework [51], using the carbon oxidation state and the saturation concentration of the pure compound to define the organic aerosol composition in a two-dimensional space. A hybrid approach of condensation/evaporation and absorptive partitioning into an organic liquid was applied to treat condensation to an organic mixture considering the non-ideal solution behavior of the organic mixture.

Biogenic secondary, anthropogenic secondary (from oxidation of aromatic and aliphatic VOC), and delayed primary organics are represented by two SOA compounds of different volatility each. SOA precursors are formed in the chemistry mechanism EmChem09-HET in the reactions of various VOCs with OH and NO_3_ radicals or O_3_. This includes reactions of the surrogate species XYL (lumped surrogate of reactive aromatic hydrocarbons), C_3_H_6_ (propene and other alkenes with >4 C atoms), *n*C_4_H_10_ (n-butane and other alkanes with >3 C atoms), isoprene, APIN (α-pinene and other relatively slow reacting monoterpenes), and LIM (limonene and other fast reacting monoterpenes). The lumped SOA products from the oxidation of XYL, C_3_H_6_ and *n*C_4_H_10_ are ASOA and ALOA (aromatic and aliphatic SOA components). The lumped SOA products from the oxidation of isoprene, APIN, and LIM are BSOA and BLOA (biogenic SOA components). In addition, two SOA components (PIOA and PSOA) represent delayed primary organics in the exhaust of ships and vehicles. Extremely low volatile SOA products are currently not considered to limit the number of SOA tracers. The various SOA model components and their estimated saturation mass concentration C^0^ (saturation concentration of vapor over a pure, sub-cooled liquid, in µg m^−3^) are given in Table S2, Supplementary Materials.

### 2.2. Meteorological Data

Meteorological data for the CMAQ model runs were provided by a simulation of the COSMO model [35] using version COSMO5-CLM16 [52]. The vertical resolution of the meteorological model output was 40 terrain-following geometric height levels up to 22 km altitude. The Meteorology-Chemistry Interface Processor (MCIP) from US EPA (https://www.epa.gov/cmaq/meteorology-chemistry-interface-processor, accessed on 10 May 2023) ingested output from the COSMO model to prepare the meteorology files that can be used within the CMAQ modeling system. Where possible, MCIP uses data directly from the meteorological model to maximize consistency with CMAQ. When the meteorological model does not explicitly output specific atmospheric fields, MCIP uses scientific algorithms to create those fields for CMAQ. High-resolution meteorology obtained from COSMO-CLM on a 0.022° × 0.022° grid resolution was used for the CMAQ simulation of the 4 km nest (southern France) and the city-scale simulation with EPISODE-CityChem. The pre-processor WRF4CC, which is included in the EPISODE-CityChem distribution, was used to adapt the COSMO meteorological data to the model grid of the city-scale modeling. In this way, an optimum consistency between the regional scale and city-scale CTM simulations was achieved. The WRF4CC pre-processor allows interpolation and adaption of meteorological output from either the Weather Research and Forecast (WRF) model [53] or the COSMO model to the city-scale CTM.

### 2.3. Emission Data

#### 2.3.1. Shipping Emissions

Shipping emissions from the Ship Traffic Emission Assessment Model (STEAM) model [54,55,56], version 3.5, were used in the regional and city-scale simulations.

The STEAM model computes vessel-specific emissions based on the Automatic Identification System (AIS) transponder data and a technical description of the world fleet. For VOC emission modeling, STEAM divides VOCs into four groups based on their emission factor behavior as a function of engine load (increasing, decreasing, constant, and quadratic). These groupings are based on earlier measurement work of VOC speciation as a function of engine load [57,58,59,60] and they allow for the determination of mass fractions of VOC emission inventories for specific compounds and make volatility-based assignments of VOC emissions possible. However, current VOC parameterization in STEAM is mostly based on heavy fuel oil (HFO) usage in 2-stroke engines, because most of the world fleet operates engines of this kind with residual fuels and may lead to significant uncertainties for 4-stroke/distillate fuel use cases.

For this work, regional and local emission inventories were prepared with STEAM using the 2020 AIS data as a baseline; therefore, including changes in the ship traffic due to the SARS-CoV2 pandemic. Existing Emission Control Area rules are built in STEAM, which requires ships to use 0.1%S fuel in the North Sea and the Baltic Sea region. For ports, EU sulfur regulation makes 0.1%S fuel use mandatory while in port areas. The regional shipping emission dataset for Europe has a spatial resolution of 36 km × 36 km and the regional emission dataset for the Mediterranean Sea has a resolution of 12 km × 12 km. Both datasets have a temporal resolution of 1 h and the emissions are divided into two vertical layers (0 to 36 m; 36 to 1000 m above ground). In CMAQ, shipping emissions were distributed in the two lowest layers, emissions below 36 m were attributed to the lowest layer, and emissions above 36 m were in the second layer [38].

The local emission inventory includes shipping activities around Marseille and in its port. The local STEAM dataset consisted of hourly emissions of major pollutants, VOC, and particle numbers from ships on a 250 m × 250 m grid resolution. The four VOC groups of STEAM were distributed between reactive VOC and primary volatile organics of the city-scale model (see Table S3, Supplementary Materials). The local shipping emissions from STEAM were projected and interpolated to the city’s model grid. Finally, the emissions of the two vertical layers were summed up since the vertical distribution of ship emission is calculated in EPISODE-CityChem, individually at every x-y position of the surface grid, generating individual exponential Gauss distributions [61] depending on the current wind speed and stability.

#### 2.3.2. Emissions of Other Sectors

The European CAMS-REG-AP (version 5.1) emission inventory for BAU (business as usual) in the year 2020, available at the Emissions of Atmospheric Compounds and Compilation of Ancillary Data (ECCAD) website (https://permalink.aeris-data.fr/CAMS-REG-AP, accessed on 10 May 2023) was used for both the regional and city-scale simulations. The CAMS-REG-AP emission inventories are provided with a grid resolution of 0.1° × 0.05°, equivalent to ca. 6 km × 6 km over central Europe [62].

For the application in CMAQ, the data were re-gridded and vertically and temporally redistributed. Sector- and country-specific temporal profiles of lockdown adjustment factors to account for emission reductions during the SARS-CoV2 pandemic lockdown were applied as in Matthias et al. [63]. The emission splits of non-methane volatile organic compounds (NMVOC) and particulate matter to the CB05 species of the CMAQ model from the year 2015 were applied as no newer splits were available.

For the application in the EPISODE-CityChem model, road traffic emissions from CAMS-REG-AP v5.1 for 2016 were scaled to 2020 for citywide emissions of Marseille based on the temporal emission development in previous years. Hourly road traffic emissions scaled with lockdown factors were produced using the UrbEm hybrid method [64]. The UrbEm software v1.1 converted the gridded road transport emissions from CAMS-REG-AP into a dataset of line sources by applying major road types of the OpenStreetMap (OSM) database. The composition of the vehicle fleet assumed a fraction of 10% heavy-duty and commercial vehicles. A NO_2_-to-NO_x_ ratio of 0.3 was applied to recalculate NO_2_ emissions because of the expected higher real-world NO_2_ emissions from diesel vehicles. Road traffic emissions of total semi-volatile organic compounds (SVOC) were estimated by applying a SVOC-to-hydrocarbon emission ratio of 0.026 [65], and then assigning each 50% to the primary intermediate and semi-volatility organic compound of EPISODE-CityChem.

In total, the emission set for road traffic included 5922 line sources in Marseille. Emissions from other sectors (energy production, residential heating, solvent use, non-road traffic, etc.) were based on the CAMS-REG-AP emission inventory for BAU (business as usual) in the year 2020, with lockdown adjustment factors on the city scale as in [63]. Emissions from CAMS-REG-AP were downscaled to the urban area using the UrbEm software v1.1, generating area sources with 500 m × 500 m grid resolution for the Marseille domain.

#### 2.3.3. Natural Emissions

CMAQ simulations considered natural emissions of atmospheric constituents. Sea salt emissions were calculated as described in Kelly et al. [66]. Biogenic VOC (BVOC) emissions from vegetation and nitrogen monoxide (NO) from soil were calculated separately with the MEGAN model v3 (Model of Emissions of Gases and Aerosols from Nature; [67,68]). Emissions of wind-blown dust were not considered.

EPISODE-CityChem simulations for Marseille considered tree-specific BVOC emissions based on a European inventory with a grid resolution of 100 m × 100 m [69], which relies on tree cover data from the Copernicus Land Monitoring Service’s (CLMS) Tree Cover Density map (TCD) [70]. The TCD map was combined with probability maps of the 39 most common tree species in Europe [71]. The CLMS Forest Type Additional Support Layers were used to identify trees in an urban context on a map with 100 m × 100 m resolution; in combination with urban-specific mixes of tree species for different bioclimatic zones. These maps were then combined with plant-specific foliar biomass density and standard emission potentials for isoprene, monoterpenes, sesquiterpenes, and oxygenated VOCs [72]. Based on this, hourly emissions of isoprene and monoterpenes were calculated depending on temperature [68] and solar radiation for the Marseille urban domain.

#### 2.3.4. Urban Particle Number Emissions

An urban emission inventory of particle numbers has been prepared for Marseille, including PN emissions from local shipping (Section 2.3.1), residential heating, and road transport sources. For residential heating, a particle number emission inventory was created based on the heating demand and fuel types used based on the city average in Marseille, and population density. Emission factors of particle number for different fuel types (solid, heating oil, natural gas) as given in Lauenburg et al. [30] were weighted for the respective fuel shares. For road transport, annual total PN emissions for all urban line sources were estimated based on vehicular NO_x_ emissions by applying a conversion factor of 2.15 × 10^14^ per gram of emitted NO_x_, as given by Ketzel et al. for workdays [73]. PN emissions from industrial combustion processes were not considered because the major industrialized area, Fos-Berre, northwest of Marseille, lies outside of the model domain. Standard particle emission size spectra, which have been determined in our previous work [30], were applied to total PN emissions from local shipping, residential heating, and road traffic in the city-scale simulations (Figure S2, Supplementary Materials).

### 2.4. Air Quality Monitoring

Measurements of hourly concentrations of O_3_, NO_2,_ and PM_2.5_ at monitoring sites of the Air Quality Monitoring Network belonging to the regional air quality agency AtmoSud for the period June to October 2020 were used to validate the model performance of EPISODE-CityChem. NO_2_ was measured with a chemiluminescence analyzer, O_3_ by a photometric analyzer, and PM_2.5_ with a Tapered Element Oscillating Microbalance equipped with a Filter Dynamic Measurement System (TEOM-FDMS, Thermo Fisher Scientific, Waltham, MA; USA). Measurement data was transmitted in real-time every 15 min by the online measuring devices. The model–observation comparison was evaluated for selected monitoring stations: (1) Marseille Longchamp (LCP; 43°18′18.94″ N; 5°23′41.32″ E), an urban background station; (2) Marseille Place Verneuil (VER; 43°18′32.20″ N; 5°22′04.66″ E), an urban traffic station; (3) Marseille Saint Louis (STL), an urban background site in proximity to the industrial area; (4) Aubagne (AUB), a suburban background station outside of the city center to the east of Marseille; and (5) Vallee de l’Huveaune (HUV), a peripheral urban industrial site. LCP is an aerosol supersite operated jointly by AtmoSud and Aix Marseille University. Air quality stations and measured pollutants are listed in Table S4, Supplementary Materials**.**

### 2.5. Measurement Campaign

**Figure 3:**
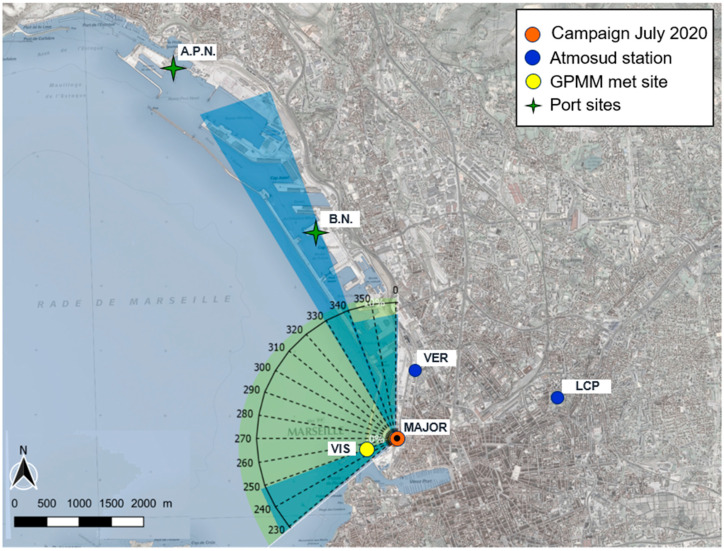
Map of the port of Marseille, with field campaign station La Major (orange filled circle), air quality monitoring sites LCP and VER (blue filled circles), and met site VIS (yellow filled circle). Two port sites are marked by green stars, B.N. (Bassin National) and A.P.N. (Avant Port Nord). Wind sector (230–360°) used for ship plume identification (winds blowing from port and sea) indicated as green shaded segment. Blue shaded segments indicate wind sectors with influence from main areas of port activities and ship lanes.

Field measurements at the site La Major (43°18′0.51″ N; 5°21′48.01″ E) in the port of Marseille (Figure 3) were conducted in the period from 3 to 20 July 2020. The campaign was part of the EU-funded H2020 project SCIPPER (Ship Contribution to Inland Pollution. Push for Enforcement of Regulations). In this period, the total average ship traffic density amounted to 23 arrivals/departures per day and mostly consisted of passenger ships (cruise ships, Ro-Ro passenger, and vehicle ferries) (source: GPMM; https://www.marseille-port.fr, accessed on 10 May 2023). The number of arrivals/departures and related ship type categories per day are shown in Figure S3, Supplementary Materials.

A mobile laboratory “Massalya” hosted online instruments for the analysis of particulate matter with aerodynamic diameter less than 1 µm (PM_1_), particle number and VOC operated during the campaign. PM_1_ chemical composition of the non-refractory species (ammonium nitrate, ammonium sulfate, ammonium chloride, and organic matter) were determined using a High-resolution Time-of-Flight Aerosol mass spectrometer (HR-ToF-AMS, Aerodyne, Billerica, MA, USA). Particle number and size distribution in the range of 14.7–673 nm were measured using a scanning mobility particle size (SMPS 3936, equipped with a CPC 3775, TSI, Aachen, Germany).

For analysis of VOC, sample air was directed to a PTR-ToF-MS (PTR-ToF-8000, Ionicon Analytik, Innsbruck, Austria) at 200 mL min^−1^ using a separate 1/16″ Silcosteel-coated tube. The PTR-MS was run at a 10 s time resolution; the AMS at 1 min and the SMPS at 2 min time resolution. The main organic molecules detected by PTR-MS during the measurement period are listed in Table S5, Supplementary Materials.

In addition, measurements of particle number and size distribution in the range of 15–650 nm with an SMPS (SMPS 3938 equipped with a CPC 3752, TSI, Aachen, Germany) at the aerosol supersite LCP, and measurements of wind speed and direction at met station VIS (operated by the port authority GPMM, see map in Figure 3) during July 2020 were used in this study. The air arriving at the site was influenced by the shipping activities of the whole port area when the mistral winds (from the northwestern wind sector) occurred. During the campaign period, winds blowing from port and sea had a frequency of 69% and winds from main shipping emission source areas had a frequency of 34%.

### 2.6. Method to Estimate Oxidative Potential

Typically, acellular assays are used to quantify particle-bound ROS and the entire OP of particulate matter, including the dithiothreitol assay (DTT), ascorbic acid assay (AA), and 2,7-dichlorofluorescin/hydrogen peroxidase assay (DCFH). Statistical methods have combined measurements of PM_2.5_ or PM_10_ concentrations and co-located acellular array responses to connect OP to certain sources of particle pollution. In this study, we estimated the OP due to particulate organic matter from shipping in Marseille. The method uses OP parameters of organic aerosol components, related to their PM_2.5_ mass, OP_m_ (where OP_m_ is the OP activity per mass of the aerosol component, in units of nmol min^−1^ μg^−1^) given in the study by Daellenbach et al. [74]. While PM_10_ concentrations in Europe are dominated by the coarse fraction (crustal material and secondary inorganic aerosols), the OP of inhaled particulate matter is dominated by organic components in the fine fraction. Thus, it is sufficient to estimate OP_v_ (OP activity per volume of air of the aerosol component, in units of nmol min^−1^ m^−3^) of inhaled ambient air based on the organic aerosol components in PM_2.5_. OP_m_ relations for different assays are displayed in Table 1.

We combined the OP_m_ of the different organic aerosol component concentrations (in PM_2.5_) with the air-quality model EPISODE-CityChem to estimate the OP_v_ that can be attributed to shipping. The OP_v_ of POM was calculated according to:

(1) $${\mathrm{OP}}_{v}\left(\mathrm{POM}\right)=\sum_{\mathrm{OA}=1}^{P}{\Delta C}_{\mathrm{ship}}(\mathrm{OA})\;\times \;{\mathrm{OP}}^{m}\left(\mathrm{OA}\right),$$

where ΔC_ship_(OA) is the spatial average of the modeled ship-related mass concentration of an organic aerosol component, OA, within the port city area (indicated in Figure 1). OP_v_(POM) is obtained by summation over the different OA (as in Table 1), and P is the total number of organic aerosol components. OP_v_(POM) was estimated for the three acellular assays, while noting that DDT is the only assay that is sensitive to primary organic aerosol from vehicle and ship emissions. The model SOA surrogates PIOA and PSOA were attributed to HOA, ASOA and ALOA to aSOA, and BSOA and BLOA to bioSOA. The organic aerosol from biomass burning was not considered as the contribution of local biomass burning can be neglected during summer in Marseille [75].

Costabile et al. [76] reported a clear association of OP_m_ measured in DCFH with the median particle diameter of the surface-area size distribution of fresh vehicular UFP emissions using principle component analysis. Unfortunately, they did not provide the belonging OP_m_ parameters that could be used here.

## 3. Results

### 3.1. Model Evaluation

#### 3.1.1. Comparison to Air Quality Monitoring Data

EPISODE-CityChem simulations with the coupled regional-to-local setup were performed for the months of June to October 2020. For validation, simulated hourly mean concentrations of NO_2_, O_3_, and PM_2.5_ were compared to available observation data from selected air quality monitoring stations (Section 2.4) in Marseille. In the statistical analysis of the model performance, the normalized mean bias (NMB), root mean square error (RMSE), Pearson correlation coefficient (r), and the fraction of predictions within a factor of two observations (FAC2) were evaluated. The statistical analysis was done with the R package “openair” [77]. Table 2 gives a summary of the performance statistics.

Pearson correlation coefficients were in the range of r = 0.23–0.38 for hourly NO_2_, r = 0.33–0.41 for hourly O_3_, and r = 0.17–0.22 for hourly PM_2.5_. Despite the poor correlation of modeled and measured PM_2.5_, the error of PM_2.5_ in terms of RMSE (5.4–7.9 µg m^−3^) is smaller than the mean RMSE from different regional scale models of 10.3 µg m^−3^ for urban sites in the AQMEII intercomparison study [78].

Comparison of measured vs. modeled hourly values show FAC2 values of 0.16–0.39 for NO_2_, 0.63–0.68 for O_3_, and 0.69–0.73 for PM_2.5_, which satisfies the acceptance criteria of FAC2 = 0.3 for urban dispersion model evaluation [79], except for NO_2_ at suburban background site AUB. High negative bias for NO_2_ (NMB = −0.75) at AUB indicates that the regional background of NO_x_ is too low, consistent with the underestimation of observed NO_2_ concentrations by CMAQ in the Mediterranean region [38]. The main reasons for the weaker performance of EPISODE-CityChem for Marseille compared to previous dispersion studies ([31,80]) for Hamburg (Germany) are the lower representativeness of the pollutant concentrations inherited from CMAQ at the boundaries and the difficulties of the meteorological model to simulate the diurnal sea/land breeze cycle.

A trend analysis of the time series of modeled and measured daily mean concentrations of NO_2_, O_3_, and PM_2.5_ was performed at all monitoring stations with available measurements during July 2020 (Figure S4, Supplementary Materials). The Mann–Kendall trend test from R-package Kendall was used to analyze the time series for monotonic trends based on the Kendall rank correlation. A weak positive trend was confirmed for both the model and observations of NO_2_ and PM_2.5_ at the traffic site VER. For the urban background site LCP, the modeled daily means of NO_2_ and PM_2.5_ showed no trend or very weak trend, while the observed daily means had a weak positive trend. For the two other sites with PM_2.5_ observations (RAB and STL), the observed trend was stronger than the modeled trend. The weaker daily trends of modeled PM_2.5_ give an indication for events of transported particulate matter from the larger region (e.g., dust events) that have not been captured by the regional air quality model.

**Figure A1:**
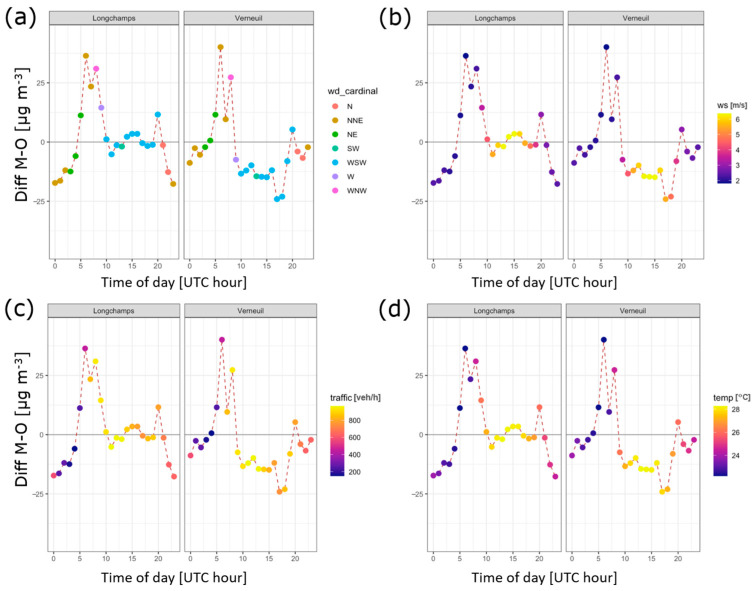
Diurnal variation of the model–observation NO_2_ concentration differences (Diff M-O in µg m^−3^) at sites LCP (**left**) and VER (**right**). Color symbols indicate (**a**) average wind direction; (**b**) average wind speed; (**c**) average traffic intensity (on workdays); and (**d**) average air temperature.

**Figure A2:**
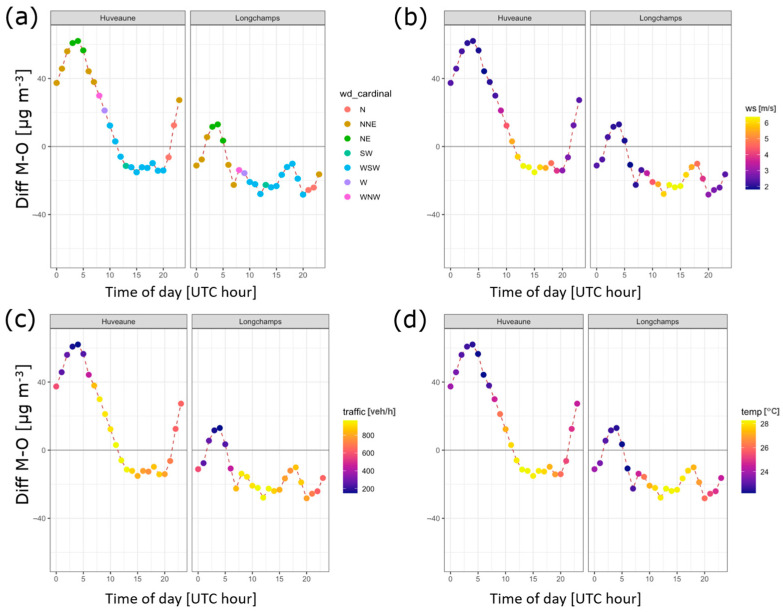
Same as in Figure A1 for model–observation O_3_ concentration differences.

**Figure A3:**
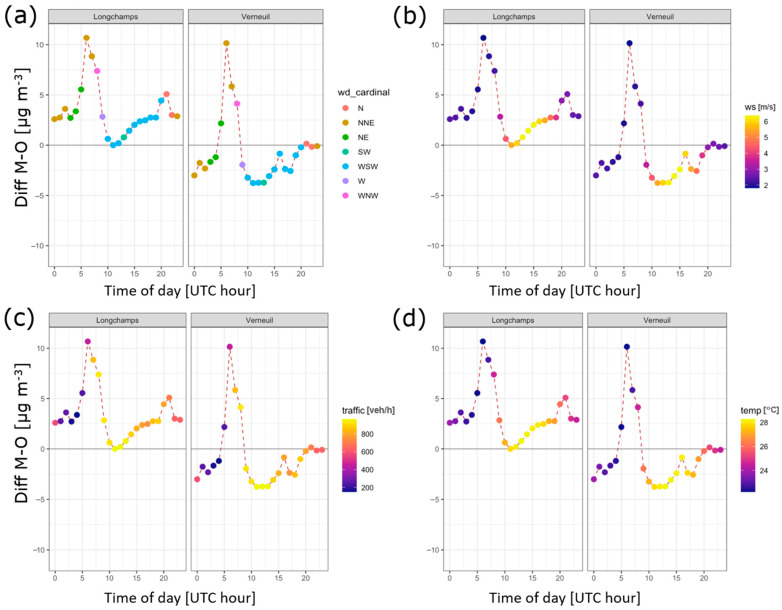
Same as in Figure A1 for model–observation PM_2.5_ concentration differences.

Further, we inspected the influence of meteorological parameters and traffic intensity on the diurnal variation of the model–observation (M-O) difference (Appendix A, Figure A1, Figure A2 and Figure A3) to find reasons for the weak correlations between modeled and observed time series in July 2020. For NO_2_ and PM_2.5_, the focus was on the traffic site VER within the port area and the site LCP, since these are the most relevant sites (Figure 3) for the investigation of ship impacts. The model strongly overestimated observed concentrations of NO_2_ and PM_2.5_ in the morning hours (5–9 a.m., UTC) at both sites, connected to winds from north to northeast and the morning rush hour. For the remaining daytime (10 a.m. to 8 p.m., UTC), during which concentrations are diluted in response to the increase in the boundary layer (average wind speed ≥ 4 m s^−1^), modeled NO_2_ concentrations were close to observations at LCP, but lower than observations at VER. The underestimated NO_2_ at the traffic site is probably because of too low vehicular emissions (traffic counts: 700–1000 vehicles per hour) or missing port emissions in the model during this time of the day. For O_3_, the focus was on LCP and the peripheral industrial site HUV. The model strongly overestimated observed O_3_ concentrations at night and in the morning at HUV, but not at LCP. During this period, characterized by land–sea wind and low wind speed (2–3 m s^−1^), O_3_ is controlled by the titration with NO that accumulated in the nocturnal boundary layer. The difficulties in simulating O_3_ at night are attributed partly to the uncertain NO_2_-to-NO_x_ ratio of vehicular emissions and partly to the difficulties of the model in representing the vertical diffusivity under stable conditions. Nevertheless, we consider EPISODE-CityChem appropriate for investigating the contribution of local ship emissions in Marseille, as M-O differences are relatively small during times when the wind is blowing from the sea.

#### 3.1.2. Particle Number Data

Monitoring of ultrafine particles is not part of the regular monitoring in Marseille. The aerosol supersite LCP gathers a complete set of unregulated pollutant measurements, including size-resolved particle number concentrations. For the comparison of modeled and measured size-resolved PN concentrations, the observation data at LCP during July 2020 was used. The performance of the model for predicting particle number concentrations was evaluated based on model–observation value pairs of hourly mean total PN concentrations (N = 561). Hourly measurements at the urban background site were mostly matched within a factor of 2 (FAC2 = 0.62) and predicted values show only a small positive bias (NMB = 0.09). The accuracy of hourly model predictions was moderate, as indicated by the root mean square error (RMSE = 11,900 cm^−3^) mainly due to the low correlation (r = 0.16). Nevertheless, the maximum of the observed monthly mean PNSD at 21–50 nm diameter and the shape of the observed number size distribution were well reproduced by the model (Figure S5, Supplementary Materials).

### 3.2. Potential Ship Impact

#### 3.2.1. Size-Resolved PN Concentrations and SOA Composition

**Figure 4:**
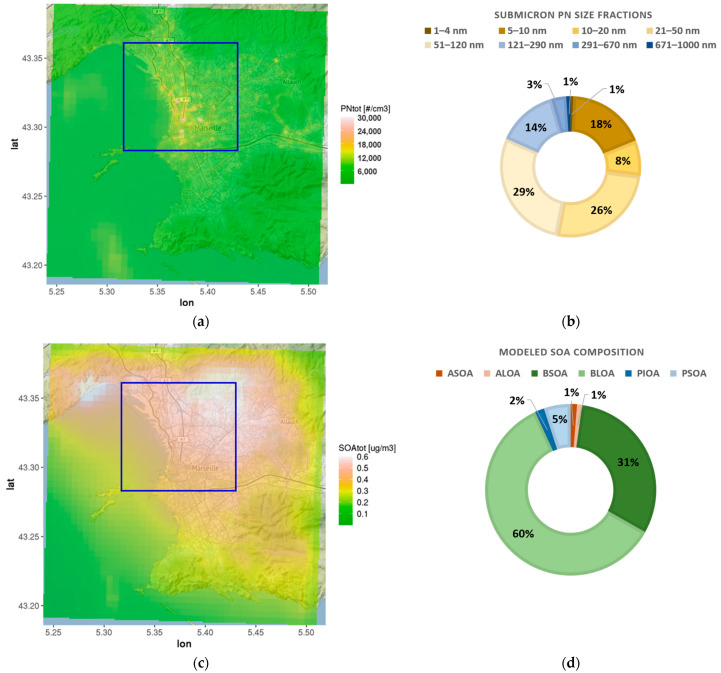
Simulated monthly mean (July 2020) concentrations of PN and SOA for Marseille: (**a**) total PN; (**b**) average size fractions of submicron PN in the city port area; (**c**) total SOA; (**d**) average SOA composition in the port city area. The extent of city port area is indicated as blue frame in figure parts (**a**,**c**).

The spatial distribution of the monthly mean (July 2020) concentrations of total PN simulated with EPISODE-CityChem reveals the highest values (2.5–3.0 × 10^4^ cm^−3^) along the road network and in the port areas (Figure 4a).

The monthly mean total PN was 6500 cm^−3^ on average within the port city area. About 80% of the particles were in the ultrafine size range, which is in accordance with previous urban studies [11,81], and 53% of the particles were smaller than 50 nm in diameter (Figure 4b). The monthly mean SOA was 0.46 µg m^−3^ on the spatial average of the port city area. High SOA concentrations were predicted in the northwestern urban area that is surrounded by a mountain range (massif de l’Etoile), indicating the influence of BVOC emissions from forests (Figure 4c). The average composition of modeled SOA reveals a very high share (91%) of biogenic SOA (Figure 4d).

High levels of non-fossil oxygenated organic aerosol during summer have been reported from a field study in Marseille that used aerosol chemical composition characterized by AMS in combination with positive matrix factorization (PMF2) to investigate sources and aging of organic aerosols [82]. Despite extensive urban and industrial emissions in Marseille, the secondary (oxygenated) organic aerosol was found to be predominantly (~80 ± 8%) from biogenic sources. According to this study [82], oxidation of monoterpenes plays a major role in the formation of biogenic SOA, with a contribution of ~40% to the non-fossil oxygenated organic aerosol.

#### 3.2.2. Ship Contributions to Total PN and SOA

We determined the potential impact of shipping by performing an additional model run with EPISODE-CityChem with all ship emissions deactivated. This additional model run is referred to as “noship” run in the following. The potential impact of local shipping (in the port and the coastal area of Marseille) was calculated as the concentration differences between the reference run (“ref”) and the “noship” run and is provided either as ship-related concentration, ΔC_ship_, or as a relative percentage value ([ΔC_ship_/C_ref_] × 100%). Table 3 summarizes the potential ship impacts of various air pollutants in terms of percentage values and absolute values (ΔC_ship_) for the average of the port city area and four port sites: La Major (field campaign site), VER (traffic site), Bassin National (central port, location of maximum impact), and Avant Port Nord (northern port).

Ship emissions have a substantial influence on modeled ambient concentrations of total PN, NO_2_, and SO_2_ in Marseille, whereas the influence of shipping on SOA and PM_2.5_ is small. In 2019, port activities were the greatest contributor to nitrogen oxide (NO_x_) emissions (31%), followed closely by traffic emissions (30%) in the urban district of Marseille (https://cigale.atmosud.org/, accessed on 6 May 2023). The number of ship calls in the port of Marseille in July 2020 was 31% lower than in July 2019, mainly due to a decline in cruise and passenger coastal ship traffic resulting from lockdown measures (https://www.emsa.europa.eu/newsroom/covid19-impact/download/6290/3836/23.html, accessed on 28 August 2023). The global sulfur cap for marine vessels came into effect in January 2020, limiting the fuel sulfur content to 0.5%, except for ships equipped with scrubbers. The fuel sulfur limits applied for the ship emissions were 0.5% for ships at sea and 0.1% for ships at berth (Section 2.3.1). Despite the reductions in sulfur emissions from ships, SO_2_ related to local shipping contributed significantly to the simulated concentrations in the port areas, with a maximum of 18%, mainly in the cruise ship area and the ferryboat (Ro-Ro) area. High ship impacts of air pollutants over the sea are found at the main shipping routes to and from Marseille and the cross-junction of the shipping routes (Figure S6, Supplementary Materials).

**Figure 5:**
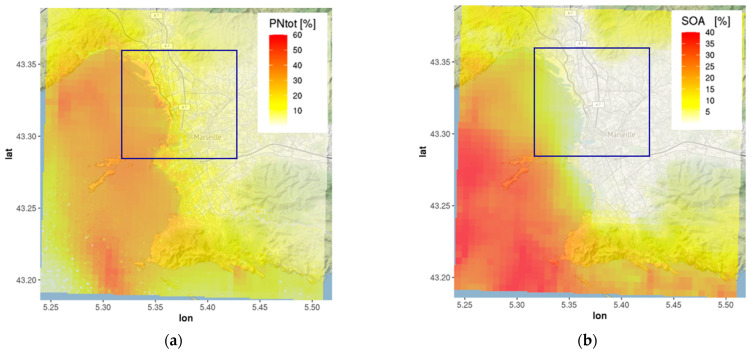
Maps of the relative potential ship impact in Marseille (monthly mean, July 2020): (**a**) potential ship impact (%) on total PN; (**b**) potential ship impact (%) on total SOA. Blue frame indicates city port area.

Figure 5 shows the spatial patterns of the potential ship impacts of total PN and SOA. The ship impact on particle number concentrations was high in port areas (27–51%) and over the coastal waters. Ship-related SOA contributed 6–9% to total SOA concentrations in the port areas, but its contribution to SOA in the inner city of Marseille was negligible. Modeled ship-related SOA was in a range of 0.03–0.04 µg m^−3^ within the port.

The reason for the low ship impact on SOA in the inner city is that modeled SOA concentrations in the city are dominated by vehicular exhaust and biogenic sources. However, the presented estimate is likely on the lower end of the potential SOA formation in ship exhaust due to missing sources of primary non-volatile organic carbon in the model that facilitate the condensation of delayed primary organics. El Haddad et al. [82] reported an average organic aerosol mass of 0.22 µg m^−3^ from industrial activities (including shipping) in Marseille during summer.

#### 3.2.3. Ship Impact on the Number Size Distribution

**Figure 6:**
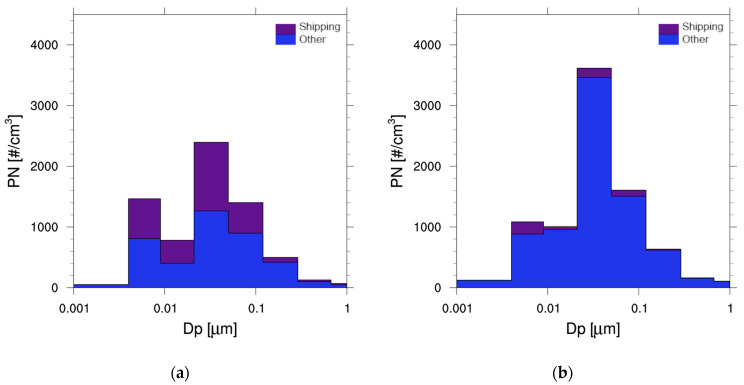
Ship contribution to the modeled size-resolved particle number concentrations (dN per size class in cm^−3^), monthly mean of July 2020: (**a**) ship-related PN (purple) and PN from other sources (blue) at campaign site La Major; (**b**) ship-related PN (purple) and PN from other sources (blue) at urban background site LCP.

The ship impact on the modeled number size distribution of particles was also examined. The ship contribution to the simulated PNSD at the campaign site La Major in the port of Marseille was substantial, with the largest contributions to particles in the ultrafine size range (Figure 6). At the urban background site LCP, at 2 km distance from the port, the contribution of ship-related particles was only minor. Road transport appears to be the dominant source of particles at the urban background site. The maximum of the ship impact on the modeled PNSD at La Major was in the size range of 21–50 nm, corresponding to the size distribution maximum of ship exhaust particles measured by Jonsson et al. [21].

### 3.3. Detection of Ship Plumes

Merico et al. [18] based on measurements of particle number and mass size distributions in two port cities of the northern Adriatic Sea (Venice, Italy and Rijeka, Croatia) have indicated that number size distributions better reflect the contribution from port emissions of ultrafine and fine particles compared to mass-based concentrations. Inspired by this finding and the simulated high ship impact on total PN concentrations and PNSD in the port of Marseille, we developed a method for ship plume detection that is based on measured and modeled PN concentrations during the field campaign (Section 2.5) at La Major. The method is described in the following.

First, measured and modeled total PN concentrations at La Major during a 1-week period (10–17 July 2020) were filtered for wind direction from sector 230–360° associated with winds blowing from the port and sea, potentially bringing ship-related particles to the site. Data during periods of stagnant wind (wind speed < 1 m s^−1^) were excluded. The measured 2 min PN concentration time series was time-synchronized to the hourly mean concentrations from the model.

Second, the predicted wind direction data (from the COSMO model) at VIS (see map in Figure 3) was used to filter the PN data. We used predicted wind direction because of data gaps in the wind measurement. The time series of predicted wind speed and wind direction were in good agreement with the measured wind data at the VIS met station (Figure S7, Supplementary Materials).

**Figure 7:**
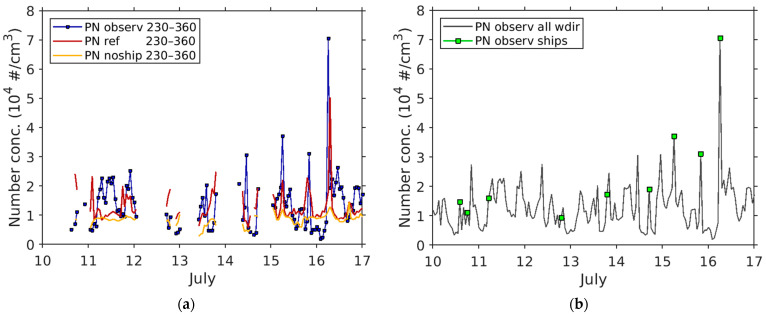
Ship plume detection during the field campaign at La Major, period of 10–17 July 2020: (**a**) time series of wind-filtered observed and simulated total PN concentration (wind sector 230–360°); (**b**) detected ship plumes in each hour of the PN observation, indicated by green-filled squares.

Third, the modeled concentration time series of total PN from the reference run and the noship run, each filtered for winds from 230–360°, were subtracted to get modeled ΔC_ship_(PN) for each hour. Figure 7a shows the wind-filtered time series of hourly PN data from measurement, reference run, and noship run.

Two criteria were then applied to detect a ship plume in the hourly data. The first criterion for the detection of a ship plume was ΔC_ship_(PN) ≥ 0.5 × C_ref_(PN) in the modeled PN time series. The second criterion was that both the observed and modeled hourly PN value at any time was 3000 cm^−3^ higher than the PN value of the previous hour.

Nine ship plumes were detected in the 1-week period using this method (Figure 7b). The new ship plume detection allows unambiguous attribution of peaks in the measured PN concentration time series to ship emissions. Although several other PN peaks were observed in the measured hourly time series, these other peaks were either not associated with wind from sea/port or linked to traffic emissions. Since the ship plume detection is based on hourly averages, it cannot be excluded that observed short-term PN peaks (5–10 min) at La Major were below the criteria for detection. No additional plumes were detected when the data for stagnant wind conditions were included.

The highest ship-related measured PN peak (hourly mean: 7.1 × 10^4^ cm^−3^) during the 1-week period was observed on 16 July 2020. On this day, the number of ship arrivals/departures was 24; the majority of these (14 vessels) were Ro-Ro passenger and vehicle vessels. Since the filtering procedure includes pollution from ships at berth (before departure) the detected PN peaks could potentially include the contribution of passenger vehicles loading for the Ro-Ro vessels [20].

### 3.4. VOC Concentrations Related to Shipping

To evaluate the simulated VOC concentrations related to shipping activities, measured and modeled concentrations at La Major were filtered for wind direction from sector 230–360°. The 10 s measurements of VOCs with PTR-MS were time-synchronized to the hourly mean concentrations of the model. The following measured individual VOCs were compared to VOCs of the model: acetaldehyde, the sum of alkenes with ≥3 C-atoms, isoprene, toluene (model surrogate XYL), butanone (model surrogate MEK), and naphthalene (model surrogate PIOC).

Table 4 presents the comparison of modeled and observed VOC concentration averages and model-to-observation (M:O) ratio for the period of 3–17 July 2020, during which VOC measurements with PTR-MS took place. With the exception of isoprene, these VOCs are emitted from ships according to the STEAM emission model (Table S3, Supplementary Materials). Observed isoprene showed little diurnal variation in contrast with its expected biogenic origin from tree emissions. The model underestimated observed isoprene on average by a factor of five, even when considering only the daytime period for the comparison. Anthropogenic sources of isoprene, linked to road traffic emissions have been reported in urban areas [83]. Based on a 13-month field campaign in Athens, Greece, Panopoulou et al. [84] reported statistically significant correlations of isoprene and monoterpenes with tracers of anthropogenic activity (e.g., carbon monoxide and black carbon), verifying that emissions from road traffic and evaporative sources (in all seasons), and heating activities (wood burning in winter), can contribute to urban isoprene and monoterpene concentrations. Toluene and butanone were on average fairly well reproduced by the model, with M:O ratios close to 1. Butanone forms in the oxidation of n-butane. Toluene and n-butane are also emitted in vehicular exhaust. Predicted concentration of alkenes and acetaldehyde, which are primarily originating from ship emissions, were much lower than their observed concentrations.

Modeled SVOC was on average four times lower than observed SVOC (measured as naphthalene). Given that C8 and C9 aromatics measured by PTR-MS might also be semi-volatile compounds, the M:O ratio of SVOC would be even lower. Together with the low-modeled concentrations of alkenes and acetaldehyde, this gives an indication for the underestimation of VOC emissions from shipping activities in the model.

### 3.5. Reconstruction of Particulate Organic Matter

A direct comparison with measurements of POM during the field campaign at La Major was not possible because the model does not include organic matter as a separate tracer. Therefore, we reconstructed the modeled particulate organic matter using simulated PM_2.5_ and SOA concentrations of the reference run as described in the following.

Based on field observations of the PM_2.5_ chemical composition in five European Mediterranean cities over a 1-year period (2011–2012), the average contribution of POM to PM_2.5_ during summer is 30% in Marseille [85]. POM consists of primary and secondary (oxygenated) organics. Measurements in the aged ship exhaust of a passenger ship showed that 66% of the organic matter in the particles is primary [86]. Thus, we assume that primary POM has a share of 20% in PM_2.5_ and apply this fraction to the modeled PM_2.5_ (POM_prim_ = 0.3 × 0.66 × PM_2.5_ = 0.2 × PM_2.5_). Further, we assume that the model underestimates ship-related SOA concentrations corresponding to the underestimation of ship-related SVOC (Section 3.4). For this reason, simulated SOA concentrations were multiplied by a factor of four. Finally, the model-reconstructed particulate organic matter, POM_rec_, was calculated as:

(2) $${\mathrm{POM}}_{\mathrm{rec}}={\mathrm{POM}}_{\mathrm{prim}}+4\times \mathrm{SOA}$$

**Figure 8:**
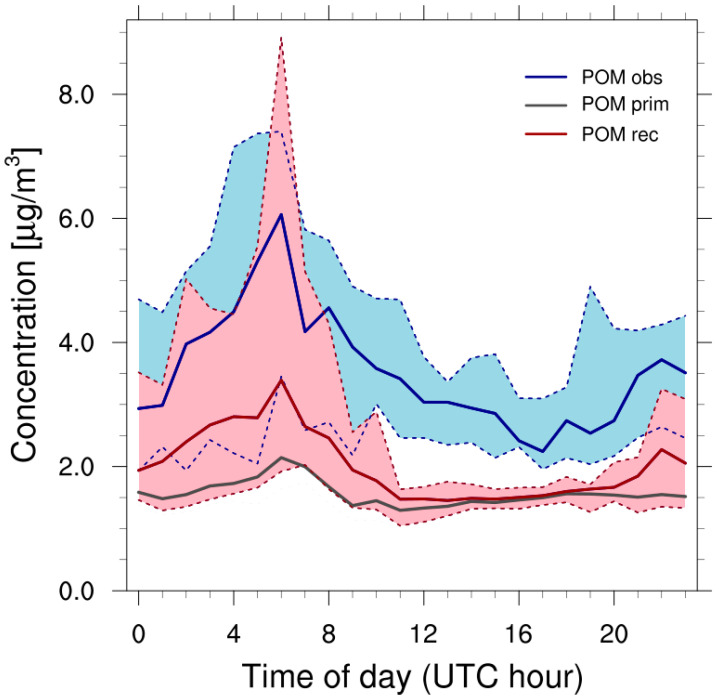
Diurnal variation of particulate organic matter (POM) as average of the field campaign at La Major (wind sector 230–360°) based on measurements with AMS (blue line) and reconstructed from modeling (red line). Grey line indicates modeled POM_prim_. Lower and upper borders of the blue and red shaded areas mark the first and third quartiles, respectively. Solid lines mark the median.

The organic matter during the field campaign at port site La Major was studied by comparing POM_rec_ from the model simulation against measurements of organic matter (in PM_1_) by AMS for the wind sector 230–360°. The daily mean observed POM (wind-filtered) was 4.1 ± 1.5 µg m^−3^ and the daily mean model-reconstructed POM was 2.9 ± 1.5 µg m^−3^ during the campaign period. The diurnal variation of measured organic matter on a campaign basis is shown in Figure S8 in the Supplementary Materials. The diurnal variation of observed and reconstructed POM shows a prominent peak in the early morning and a second lower peak in the late evening (Figure 8).

From the difference between reconstructed and primary POM, it can be inferred that SOA formation happens in the evening (during land breeze), probably as a result of photochemical processing, and then accumulates in the shallow nocturnal boundary layer. The early morning peak might be associated with the arrival/departure of passenger and vehicle carrier ships at 5–6 a.m. UTC (Figure S3c, Supplementary Materials) and with polluted air masses transported from the sea during sea breeze. Hourly POM_rec_ concentrations during the day were almost constantly about 1 µg m^−3^ below observed POM, which might imply a higher fraction of primary organic matter. Another reason for the discrepancy might be that the model did not account for the transport of SOA from the larger region to Marseille.

### 3.6. Oxidative Potential

The oxidative potential of daily mean POM related to shipping, ΔC_ship_(POM), for the average of the port city area and the port of Marseille (see Section 3.2.2) was calculated using Equation (1) and the OP parameters from Table 1. Ship-related POM_prim_ was calculated using a fraction of 0.615 in PM_2.5_ based on the average POM_prim_-to-PM_2.5_ ratio in the local shipping emissions for July 2020 from STEAM, while multiplying ship-related SOA concentrations by four due to underestimations of SVOC. Simulated POM_prim_ and primary delayed organics were attributed to HOA (hydrocarbon-like organic aerosol). The oxidative potential of ship-related POM daily means, OP_v_(POM), in the port of Marseille, was estimated for AA and DTT assays to be 0.004–0.009 nmol min^−1^ m^−3^ (city average: 0.003 ± 0.002 nmol min^−1^ m^−3^) and 0.10–0.19 nmol min^−1^ m^−3^ (city average: 0.07 ± 0.04 nmol min^−1^ m^−3^), respectively. Derived OP_v_^DCFH^(POM) was very low: 0.002 and 0.004 nmol H_2_O_2_ min^−1^ m^−3^ for city average and port maximum, respectively.

In a year-round field study, Weber et al. [87] measured OP by AA and DTT on PM_10_ filter samples at 14 different locations in France and combined the resulting OP responses with chemical speciation of PM_10_ to assess the relevance of different PM_10_ sources for the oxidative potential in Western Europe. The study identified a HFO source in Marseille attributable to port activities (for the year 2015), which presented an intrinsic oxidative potential (OP per microgram of particulate matter, i.e., OP_m_) of 0.04 ± 0.02 nmol min^−1^ μg^−1^ for AA and 0.51 ± 0.14 nmol min^−1^ μg^−1^ for DTT. Using the mean intrinsic OP values of the HFO source of Weber et al. together with the simulated ship-related POM of our study, the exact same values for OP_v_^AA^ are obtained for the port city area and the port maximum, while values for OP_v_^DTT^ are about 40% lower. Compared to other PM_10_ sources, Weber et al. [87] concluded that the HFO source is the second most important contributor to daily averages of OP_v_^DTT^ and the fourth highest contributor of OP_v_^AA^ in Marseille. According to their investigation, PM_10_ from the HFO source represented a daily mean contribution of OP_v_^DTT^ = 0.41 nmol min^−1^ m^−3^ (source: http://getopstandop.u-ga.fr/results?component=op_contrib, accessed on 22 June 2023). Compared to this, the contribution of particulate matter from shipping to daily mean OP estimated in our study is 50–75% lower. Due to the mandatory use of 0.1%S fuel at berth in European port areas, the share of HFO usage was only 2% for the vessels in the port of Marseille in 2020, according to the STEAM model. An estimate of the proportion of fuel type used by ships during stopover in the port of Marseille revealed that the vast majority of vessels (98%) in 2020 used marine gas oil (MGO) or electricity while docked.

We postulate that the lower oxidative potential in this study is connected to the use of low-sulfur fuels during stopover. For cross-validation of the postulated reduction of the oxidative potential in response to more stringent fuel sulfur regulations, relevant source apportionment studies of the last 10 years were reviewed and results were compiled in Table 5. Despite the limited number of studies that investigated the oxidative potential related to particulate matter from the marine sector using DTT assay, the review revealed that OP_v_^DTT^ values were in the range of 0.3–0.5 nmol min^−1^ m^−3^ for all ports where the former global 1.0%S limit applied to ships at sea. The only other study conducted under strict fuel sulfur regulation, in the Greater Los Angeles Area [88], found a similar low OP_v_^DTT^ value range (0.07–0.15 nmol min^−1^ m^−3^) as in our study.

## 4. Discussion

### 4.1. Uncertainties of SOA Modeling on City Scale

#### 4.1.1. Vertical Distribution and Photochemical Aging

**Figure A4:**
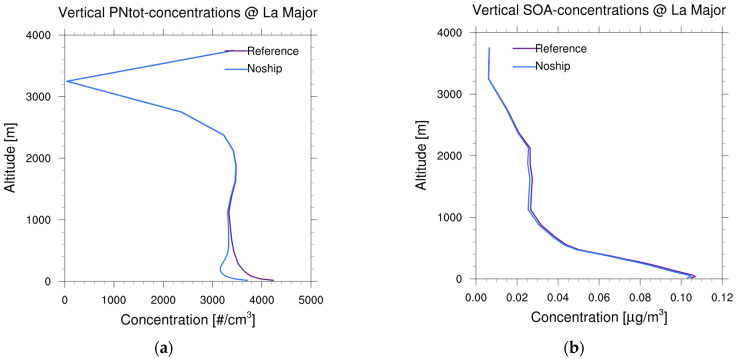
Vertical profile of (**a**) total PN and (**b**) total SOA over port site La Major as monthly mean of July 2020. The difference between the vertical profile of the reference simulation (blue line) and the “noship” simulation (purple line) corresponds to the ship’s impact on the pollutant’s profile.

Photochemical processing of shipping emissions advected to Marseille from the sea during periods of sea breeze (early morning) induces the formation of fresh secondary pollutants, such as SOA (Figure 8) and ozone, which are during the course of the day diluted with increase in the boundary layer height and/or transported outside of Marseille. Vertical profiles of SOA and total PN concentrations at La Major from the simulation in July 2020 reveal that particle numbers are impacted by ship emissions up to a height of 1000 m above ground (Figure A4, Appendix B). Despite the small contribution of ship-related SOA to the total SOA concentration, it is possible to detect the influence of shipping in the free troposphere (1000–2000 m height) over La Major. This makes it likely that air masses containing ship-related SOA and PN will be carried over long distances during which organic aerosols may undergo photochemical aging.

Observations in Marseille during summer suggest that aged air masses contain high fractions of low-volatile SOA from biogenic sources that form in the oxidative aging of semi-volatile biogenic organics over timescales of 10–20 h [82]. EPISODE-CityChem model considers the oxidative aging of primary delayed organics by OH radicals, by conversion of intermediate to low volatility products, approximated with at a first-order rate constant of 2 × 10^−11^ cm^3^ s^−1^ [91]. Liu et al. [92] observed elevated ratios of low-volatile to semi-volatile SOA near ports of the East China Sea, which indicates that port activities affected the oxidation degree of organic aerosol. The importance of aging in ship exhaust plumes was also found in a study by Pey et al. [93], who observed that aged ship emissions (identified as residual fuel oil combustion factor) prevailed over primary ship emissions in the port environment of Barcelona, Spain. This illustrates the need for more studies on the role of aging processes in coastal environments, where the oxidative capacity of the atmosphere is high and local winds frequently govern the atmospheric dynamics.

#### 4.1.2. Influence of Temperature on SOA Formation

Air temperature influences SOA formation in several ways: it affects the rate constants of the VOC oxidation, the vapor pressure of oxidation products, as well as the SOA formation mechanism and aerosol mass yields [94,95]. At high temperatures, the reaction rates will normally increase and, consequently, the formation of oxidation products will be higher than at lower temperatures. However, high temperatures will increase the evaporation of the existing semi-volatile oxidation products. The temperature dependence of saturation concentrations, $C^{0}$, of semi-volatile oxidation products in EPISODE-CityChem is approximated to first order by an Arrhenius-type equation [96]:

(3) $$C^{0}\left(T\right)=C^{0}\left(300\right)\cdot exp\left[\frac{{∆H}_{vap}}{R}\left(\frac{1}{300}-\frac{1}{T}\right)\right],$$

where R is the universal gas constant, T is the air temperature, and ${∆H}_{vap}$ is the enthalpy of vaporization. The effect of a temperature change is to shift the $C^{0}$ values of the volatility bins. The enthalpy of vaporization is critical for the strength of the temperature dependence. In global 3D CTM simulations, Tsigaridis and Kanakidou [97] investigated the influence of different values of ${∆H}_{vap}$ (42 kJ mol^−1^ and 79 kJ mol^−1^), and found the highest sensitivity to changes of ${∆H}_{vap}$ at high altitudes where the temperature is low.

**Figure A5:**
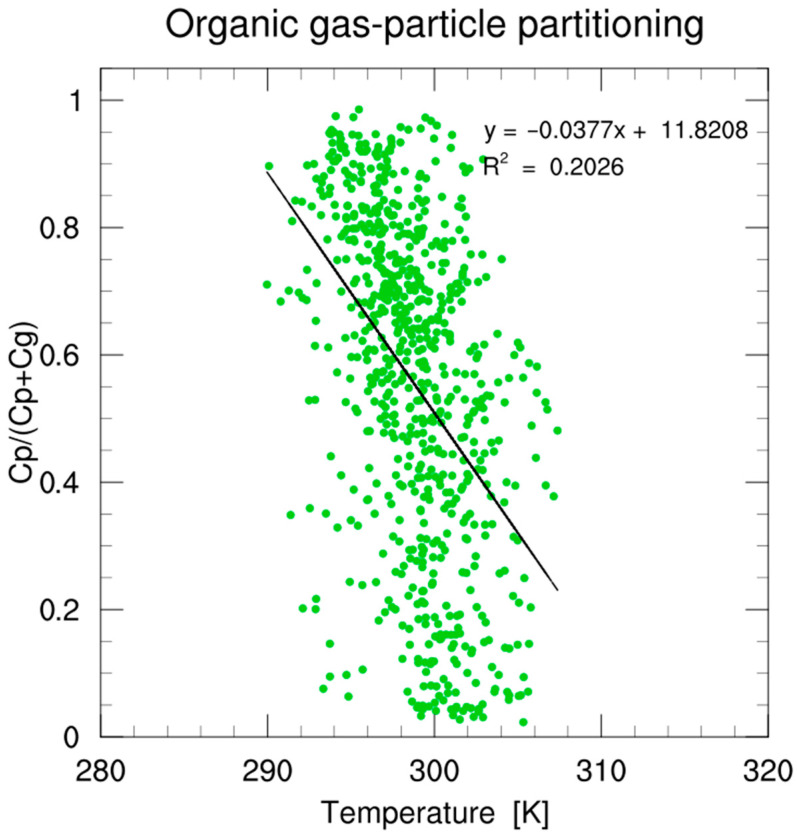
Temperature dependence of the fraction of semi-volatile organics in the particle phase (calculated as C_soc, particle_/[C_soc, gas_ + C_soc, particle_]) in the EPISODE-CityChem simulation of July 2020 (port site La Major, hourly data).

The gas-particle partitioning of simulated secondary organic products for the conditions of Marseille in July 2020 showed an approximately linear dependence on ambient temperature (Figure A5, Appendix B). High temperatures in summer favored the evaporation of semi-volatile organics. Most of the semi-volatile organics were in the particle phase at the lowest observed temperature (T = 292 K), whereas the fraction that remains in particles generally decreased with increasing temperature.

Different formation mechanisms may prevail under different temperature conditions, affecting the mass yield of SOA formation. For instance, Li et al. [98] reported increased oligomerization of n-dodecane SOA under low-temperature conditions, also modifying the optical properties of the aerosol particles. On the other hand, SOA formation from isoprene in photo-oxidation and dark ozonolysis chamber experiments revealed that increasing ambient temperature leads to decreased mass yields and the formed SOA is less volatile, containing more oligomer-like products of higher density [99].

#### 4.1.3. Limitations of SOA and UFP Modeling

In addition to the uncertainties related to oxidative aging and the temperature-dependent formation mechanism discussed above, several limitations of the current modeling approach affect the simulated SOA concentrations.

We show that the formation of ship-related SOA happens at short distances near the shipping activities at sea and in port (Figure 5). The abundance of ship-related SVOC (Figure S6d, Supplementary Materials) was highest in the areas where SOA formation occurred, which indicates that volatile organics are mainly in the gas phase because their condensation is limited by available pre-existing particle surfaces. Simulated UFP number concentrations over the sea of the coastal region of Marseille were on average 60% lower than UFP concentrations in the city of Marseille. Therefore, several factors might cause an underestimation of ship-related SOA in this study, including temperature dependence of the gas-particle partitioning, missing sources of non-volatile primary organics, and insufficient emissions of PN and SVOC from ship traffic. However, the quantitative agreement of modeled and observed total PN at the aerosol supersite provides confidence that ship emissions of PN were in the correct order of magnitude.

New particle formation (NPF) episodes in urban environments have been demonstrated to be a relevant source of UFP in cities situated in high-insolation regions [100]. Two different NPF types influencing the urban environment are discussed in the literature: (1) regional scale nucleation events, and (2) localized urban nucleation events [101]. While it would be possible to capture the regional scale NPF by prescribing the observed PNSD as a boundary condition to the city domain in the model framework of this study, local nucleation events would require the implementation of a mechanism for the nucleation of gaseous precursors (e.g., H_2_SO_4_, ammonia, and organics) in the city scale CTM. Unfortunately, the atmospheric nucleation mechanism is still surrounded by large uncertainties [49].

### 4.2. Health Consequences of Ship-Related Particles

The estimated oxidative potential for the port of Marseille (OP_v_^DTT^: 0.10 to 0.19 nmol min^−1^ m^−3^) suggests that primary and secondary organic aerosol from shipping can be significant for health effects in the neighboring area of the port. Previous studies have reported that particulate matter originating from the marine sector may significantly contribute to the oxidative potential in port cities [23,89]. Emissions related to port activities (including locomotives and trucks) accounted for 16% of the overall oxidative potential of particulate matter in the mass fraction below 250 nm diameter in the Ports of Los Angeles and Long Beach, USA [102]. Primary emitted particles from ships containing transition metals [4,19] and soot [25,103] as a result of the combustion of crude oil may be responsible for a large part of the OP related to the HFO source. However, the complexities of the chemical composition of particulate matter make it very difficult to attribute ROS activity to any specific aerosol constituent [104]. In contrast to DTT and AA arrays, which give a measure of the ability of particulate matter to generate ROS, the DCHF assay gives a measure of the ROS absorbed upon the particle surface [105]. It is interesting to note that Costabile et al. [76] found no association between the biological responses of the DHCF assay and the conventionally used metrics (i.e., PM_2.5_, soot mass, and total PN) for ultrafine particles generated from biomass burning and fossil fuel combustion. The aerosol metric most correlated to fossil fuel UFP was the median diameter of the surface-area size distribution, consistent with results from Gualtieri et al. [106] on in vitro measured biological pro-inflammatory responses.

## 5. Conclusions

Shipping represents an important source of ultrafine particles in European port cities, potentially affecting the health of residents living in neighborhoods of the harbor. We applied a coupled CTM system using ship emission data of the STEAM model on regional and local scales for estimating the impact of shipping on ambient concentrations of ultrafine particles and secondary organic aerosol in the Mediterranean port city Marseille. The city scale model reproduced the maximum and the shape of the observed monthly mean number size distribution at an urban background site. Photochemical processing of ship emissions during periods of sea breeze induced the formation of fresh secondary organic aerosol. The simulation of secondary organic aerosol derived from shipping activities involves uncertainties associated with the temperature dependence of the gas-particle partitioning, oxidative aging processes, and the amount of organic compounds emitted in ship exhaust. We draw the following conclusions:

1. Particle emissions from ships in terms of number have a profound impact in Marseille. Shipping contributes on average 27–51% to total PN concentrations in the port area. This confirms a previous PN dispersion model study that found a significant influence on shipping and port activities in Helsinki, Oslo, Rotterdam, and Athens [107].
2. Ship-related secondary organic aerosol has a low impact on simulated SOA mass concentrations in the port, partly due to an underestimation of the amounts of semi-volatile VOC emitted from ship traffic. It has been reported that the fuel shift from high-sulfur residual fuel oil to low-sulfur diesel or heavy oils tends to increase VOC emissions from ships [28].
3. Previous studies found a high relevance of port activities and shipping (HFO source) on the oxidative potential in Marseille [87] and other port cities [88,89]. In this study, the estimated oxidative potential (DTT assay) of daily mean particulate organic matter related to shipping activities, reconstructed from simulated PM_2.5_ and SOA, was 0.10–0.19 nmol min^−1^ m^−3^ in the port, lower than the reported oxidative potential of the HFO source in the study by Weber et al. [87]. The lower oxidative potential of shipping activities in our study is very likely due to the low share of ships using HFO as fuel in the port of Marseille in 2020.
4. A new method of ship plume detection was developed based on modeled and measured total particle numbers. Beyond this work, the detected ship plumes might be used for further analysis (e.g., comparing arrivals/departures of the vessels and observed VOC concentrations at the time of day when detected peaks took place) to find associations between VOCs and certain ship categories.
5. Future city-scale simulation of secondary organic aerosols should be refined by considering primary emissions of organic matter in the urban area and long-range transport of secondary organic aerosols that formed in the larger region.

Overall, the high-resolution chemistry transport model system appeared to predict both citywide and port-level concentrations of ship-related pollutants and their spatial distributions with reasonable accuracy, which may guide the development of scientifically based control policies to mitigate ultrafine particle pollution along with its associated health impacts. The relevance of particle surface area for health effects has been demonstrated in epidemiological studies [108]. The combination of measurements of particle size distributions and measurements of oxidative potential with the DCFH assay [76] in different particle size fractions together with source apportionment using EPISODE-CityChem may provide a promising opportunity for attributing health effects of ultrafine particles to certain emission sources in cities.

## Figures and Tables

**Table 1:** OP_m_ parameters of different aerosol components from DDT, AA, and DCFH assays. Entries with—indicate no significant influence. OP_m_ for AA and DTT is in units nmol min^−1^ μg^−1^ and for DCFH in units nmol H_2_O_2_ min^−1^ μg^−1^).

Aerosol Component ^1^	AA	DTT	DCFH	Reference
HOA	—	0.94	—	[74]
aSOA	0.42	0.44	0.17	[74]
bioSOA	—	0.15	0.03	[74]
BBOA	0.06	0.08	0.06	[74]

^1^ HOA: primary organic aerosol from vehicle and ship emissions; aSOA: anthropogenic SOA; bioSOA: biogenic SOA; BBOA: biomass burning in winter.

**Table 2:** Performance statistics of EPISODE-CityChem simulations at selected sites in Marseille for the period of June–October 2020 based on hourly modeled and measured concentrations.

Monitoring Site	N	FAC2	r	NMB	RMSE (µg m^−3^)
**NO_2_**					
LCP	3630	0.39	0.23	0.02	37.14
VER	3649	0.30	0.25	−0.18	44.05
AUB	3521	0.16	0.38	−0.75	12.90
**O_3_**					
LCP	3617	0.63	0.44	−0.28	32.37
HUV	3607	0.64	0.31	0.18	38.09
AUB	3532	0.68	0.31	0.09	37.87
**PM_2.5_**					
LCP	3629	0.69	0.17	0.39	7.44
VER	3656	0.73	0.22	−0.20	7.90
STL	3668	0.69	0.20	−0.22	5.41

**Table 3:** Average ship impact of various air pollutants (given as percentage contribution of shipping) in the port city area of Marseille and ship impact at four sites in the port. Absolute ship impacts (ΔC_ship_) are given in round brackets.

Pollutant	Port City Area	Port Sites			
		La Major	VER	Bassin National	Avant Port Nord
Total PN	17% (1100 cm^−3^)	43% (1600 cm^−3^)	27% (1400 cm^−3^)	51% (2300 cm^−3^)	37% (1100 cm^−3^)
SOA	2.0% (0.009 µg m^−3^)	7.3% (0.027 µg m^−3^)	6.0% (0.027 µg m^−3^)	8.6% (0.038 µg m^−3^)	6.3% (0.028 µg m^−3^)
PM_2.5_	0.6% (0.051 µg m^−3^)	2.1% (0.166 µg m^−3^)	1.0% (0.096 µg m^−3^)	1.6% (0.125 µg m^−3^)	1.0% (0.075 µg m^−3^)
NO_2_	11% (1.365 µg m^−3^)	24% (4.148 µg m^−3^)	10% (1.583 µg m^−3^)	20% (3.976 µg m^−3^)	26% (2.976 µg m^−3^)
SO_2_	8.8% (0.212 µg m^−3^)	21% (0.483 µg m^−3^)	16% (0.365 µg m^−3^)	18% (0.527 µg m^−3^)	7.8% (0.299 µg m^−3^)

**Table 4:** Comparison of modeled and observed VOC concentrations during the field campaign at La Major. Mean concentrations and standard deviation in the period of 3–17 July 2020; data filtered for wind direction 230–360°.

VOC	Modeled Conc. (µg m^−3^)	Observed Conc. (µg m^−3^)	M:O Ratio
Acetaldehyde	0.16 ± 0.05	3.13 ± 0.16	0.050
Sum of alkenes (with ≥3 C atoms)	0.10 ± 0.20	4.13 ± 4.61	0.025
Toluene (XYL)	0.76 ± 1.00	0.66 ± 1.13	1.160
Butanone (MEK)	0.32 ± 0.35	0.49 ± 0.22	0.644
Isoprene ^1^	0.11 ± 0.17	0.51 ± 0.87	0.209
SVOC ^2^	0.03 ± 0.04	0.11 ± 0.07	0.243

^1^ Isoprene as daytime mean (between sunrise and sunset). ^2^ SVOC measured as naphthalene, modeled as PIOC.

**Table 5:** Comparison of oxidative potential of particulate matter related to the marine sector, assessed by DTT assay, for different port cities of the world.

Port City	OP_v_^DTT^ Related to the Marine Sector (nmol min^−1^ m^−3^)	Observation Period	Fuel Sulfur Regulation at Sea	Reference
Marseille (France)	0.10–0.19 Daily mean in PM_2.5_	July 2020	0.5%S (global regulation)	This study
Marseille (France)	0.41 Daily mean in PM_10_	January 2015 to January 2016	1%S (global regulation)	[87]
Port-de-Bouc (France)	0.30 Daily mean in PM_10_	June 2014 to May 2015	1%S (global regulation)	[87]
Bangkok (Thailand)	0.35 ± 0.10 Daily mean in TSP	January 2016 to January 2017	1%S (global regulation)	[89]
Ningbo-Zhoushan, YRD (China)	0.53 Annual mean in PM_2.5_	October 2017 to August 2018	1%S (global regulation)	[90]
Greater Los Angeles Area, CA (USA)	0.07–0.15 Seasonal mean in PM_2.5_	September 2019 to February 2020	California: 0.1%S (within 24 nmi)	[88]

## Data Availability

The model output data and the measurement data used in this study will be made available upon request.

## Supplementary Materials

The following supporting information can be downloaded at: https://www.mdpi.com/article/10.3390/toxics11090771/s1, Figure S1: block structure of the Eulerian grid solver with P8P + 2 and the MAFOR-SOA module in EPISODE-CityChem v1.7, Figure S2: normalized size fractions (in percentage) of particles in the standard number size distributions from: (a) shipping emission; (b) residential heating emission; (c) road traffic emission; and (d) regional background, Figure S3: ship traffic at the port of Marseille during the field campaign, Figure S4: comparison of daily trends and mean concentrations of regulated air pollutants at monitoring stations in Marseille during July 2020, Figure S5: comparison of the monthly mean (July 2020) size-resolved particle number concentrations at aerosol supersite Marseille Longchamps, Figure S6: maps of the relative potential ship impact in Marseille (monthly mean, July 2020), Figure S7: comparison of predicted and measured wind data at met station VIS in the period 10–17 July 2020, Figure S8: diurnal variation (diel cycle) of organic matter in PM_1_ measured with AMS on average of the field campaign at La Major in the period 3 to 20 July 2020, Table S1: particle number (PN) parameterization scheme P8P + 2 in EPISODE-CityChem v1.7, Table S2: SOA model components and their estimated saturation mass concentration and enthalpy of vaporization used in EPISODE-CityChem v1.7, Table S3: split of the four VOC groups of the STEAM model to the VOC species (surrogates) of the EPISODE-CityChem model v1.7 given as mass fractions in the emissions, Table S4: monitoring stations of the Air Quality Monitoring Network of Marseille and measured air pollutants, Table S5: main organic molecules detected by PTR-ToF-MS during the field campaign at La Major, in the port of Marseille. The EPISODE-CityChem model v1.7 is available at https://doi.org/10.5281/zenodo.7410085, accessed on 10 May 2023.
