# Supplementary material for: Measurement and Modeling of Ship-Related Ultrafine Particles and Secondary Organic Aerosols in a Mediterranean Port City
Source: Toxics. 2023 Sep 11;11(9):771. doi: 10.3390/toxics11090771 (PMC10535743; doi:10.3390/toxics11090771)
Supplement: Supplementary file 1 [file toxics-11-00771-s001.zip › toxics-2569312-supplementary.pdf]

toxics

Supplementary materials

Measurement and modeling of ship-related ultrafine particles and secondary organic aerosols in a Mediterranean port city

Matthias Karl [1,*], Martin Otto Paul Ramacher [1], Sonia Oppo [2], Ludovic Lanzi [2], Elisa Majamäki [3], Jukka-Pekka Jalkanen [3], Grazia Maria Lanzafame [4], Brice Temime-Roussel [4], Lise Le Berre [4] and Barbara D'Anna [4]

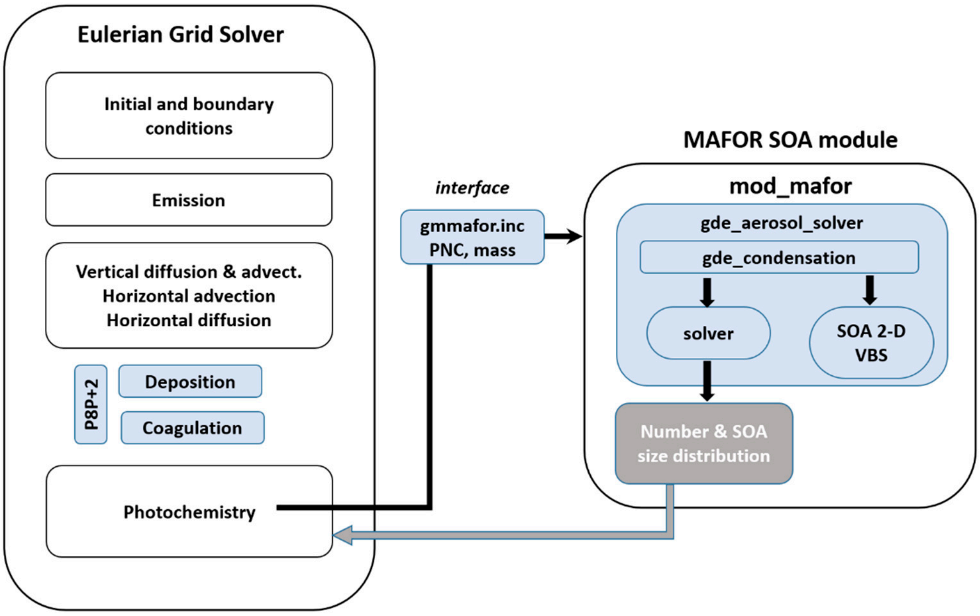

Figure S1. Block structure of the Eulerian grid solver with P8P+2 and the MAFOR-SOA module in EPISODE-CityChem v1.7. The new modules are in blue shaded blocks. The grid photochemistry calls the aerosol dynamics solver (gde_aerosol_solver) of the MAFOR-SOA module after the gas-phase chemistry is solved and updated concentrations of SOA precursor gases and sulfuric acid are available. The SOA mass concentrations are distributed over the 8 size bins below 1 μm diameter with a default distribution in the interface gmmafor.inc before the call to gde_aerosol_solver.

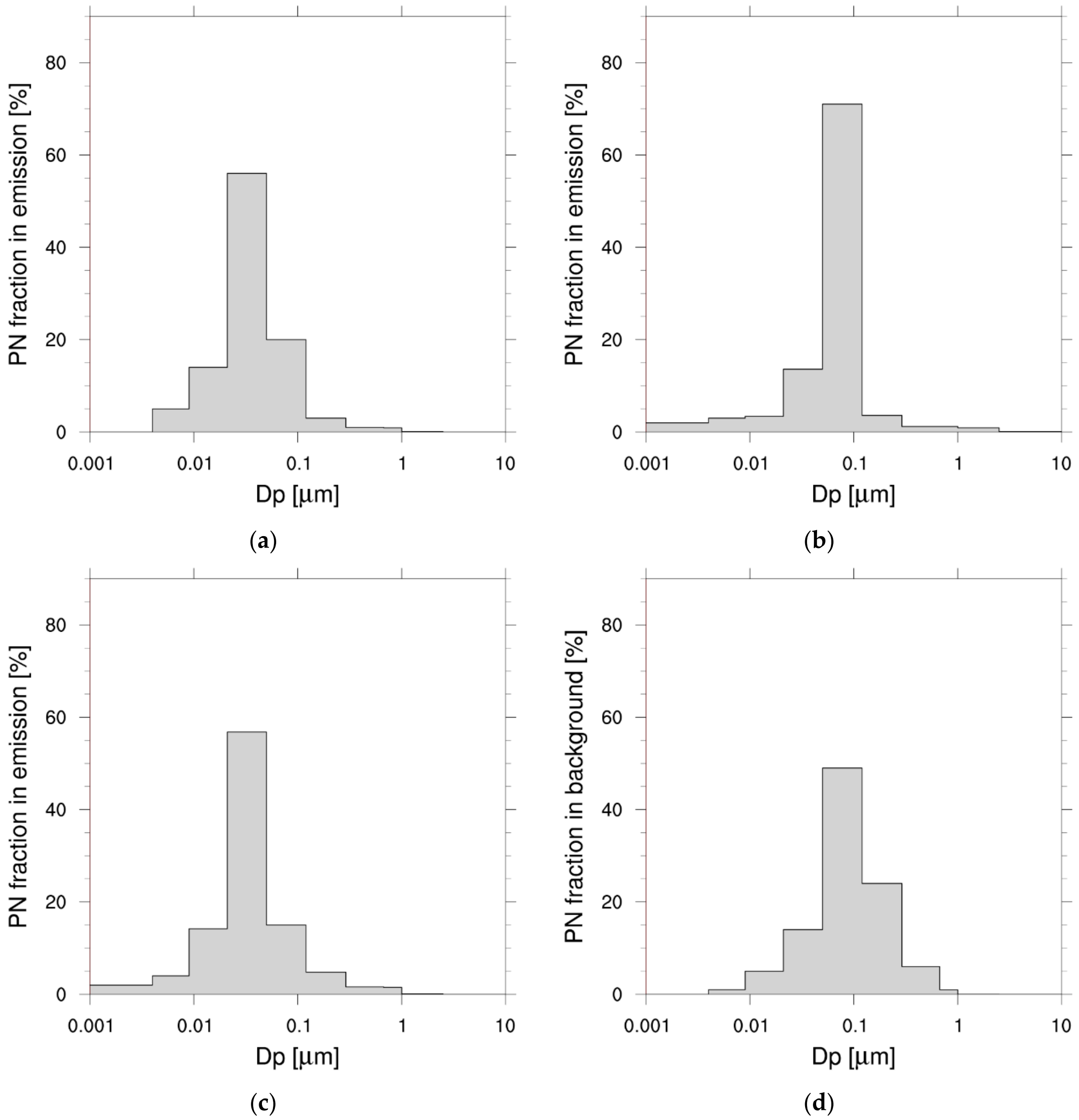

Figure S2. Normalized size fractions (in percentage) of particles in the standard number size distributions from (**a**) shipping emission; (**b**) residential heating emission; (**c**) road traffic emission; and (**d**) regional background. The x-axis presents dry particle diameter (in µm) on a logarithmic scale. The standard number size distributions were applied to translate total PN to the 10 size classes of the P8P+2 scheme in EPISODE-CityChem.

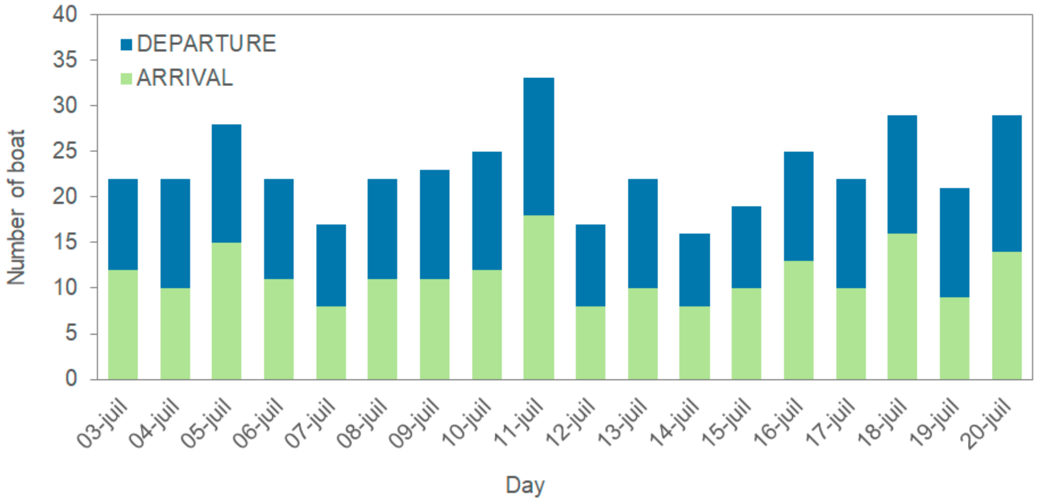

(**a**)

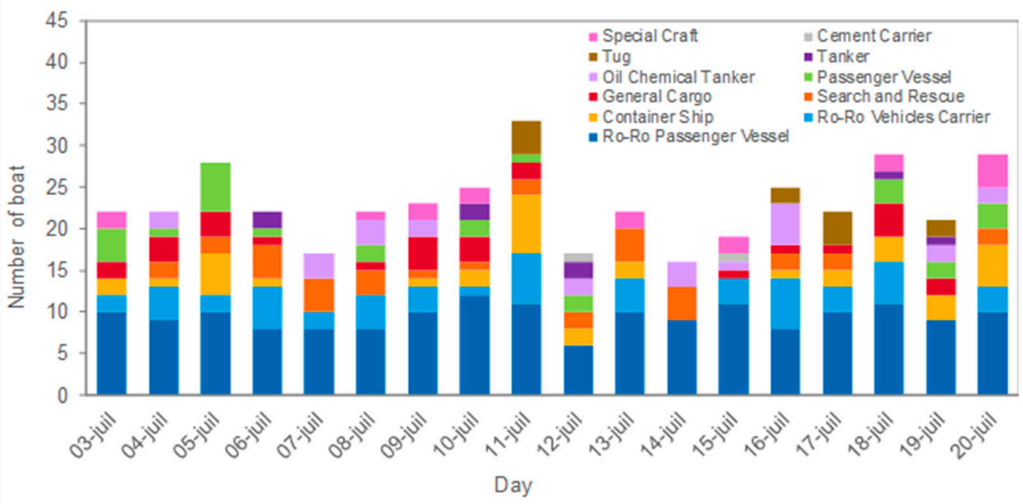

(**b**)

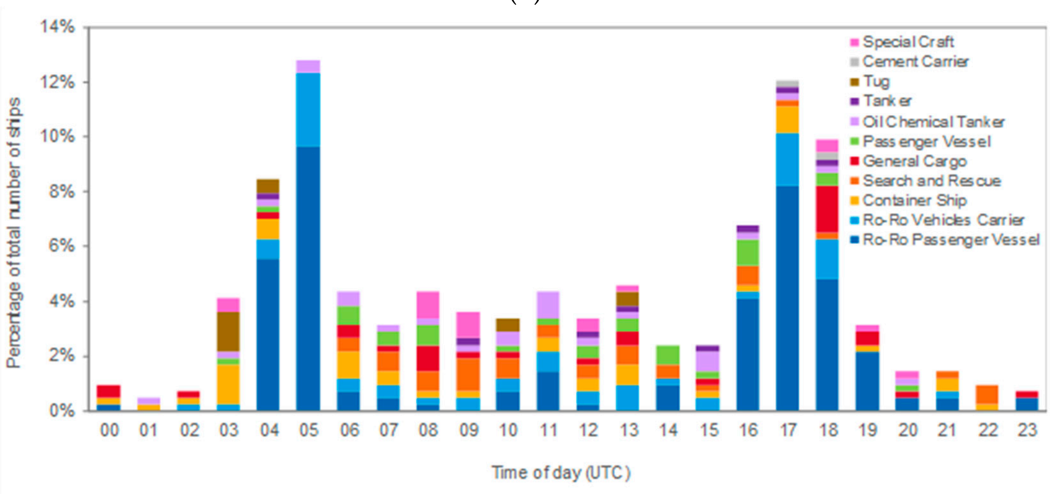

(**c**)

Figure S3. Ship traffic at the port of Marseille during the field campaign: (**a**) number of vessels with arrival and departure per day (03 to 20 July 2020); (**b**) number of arriving/departing vessels per day

divided into ship categories (03 to 20 July 2020); and (**c**) daily profile of ship arrival/departure according to ship categories, given as percentage fraction of the total number of ships during the campaign.

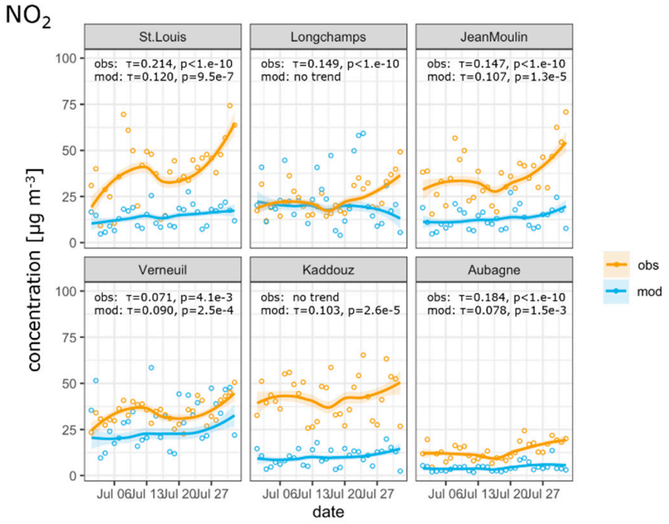

(**a**)

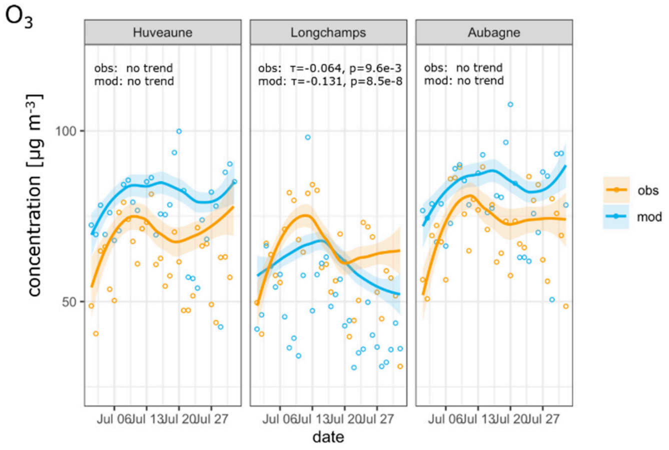

(**b**)

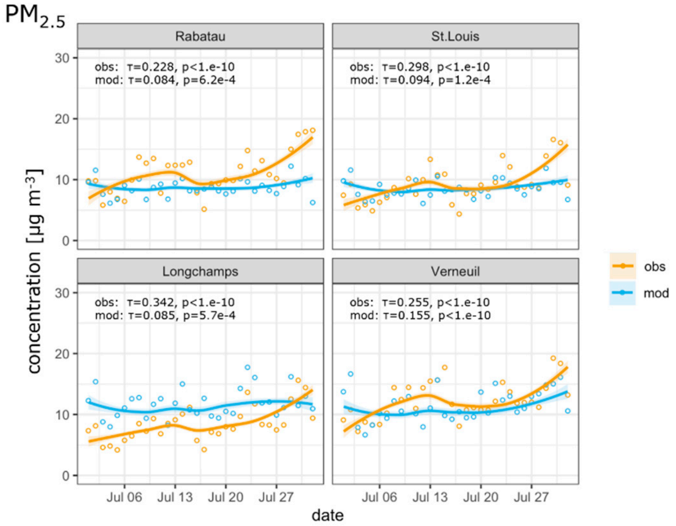

(**c**)

Figure S4. Comparison of daily trends and mean concentrations of regulated air pollutants at monitoring stations in Marseille during July 2020: (**a**) observed and modeled NO_2; (**b**) observed and modeled O_3; and (**c**) observed and modeled $PM_{2.5}$. Trend lines for modeled and observed daily means are shown with standard deviations as a shadowed range. Mann–Kendall trend test parameters (Kendall's τ and two-sided p-value) are given in the plots if the null hypothesis was rejected with 95% confidence (otherwise "no trend"). Monitoring stations: Longchamps (LCP), Verneuil (VER), Rabatau (RAB), Saint Louis (STL), Jean Moulin (JMN), Kaddouz (KAD), Huveaune (HUV), and Aubagne (AUB).

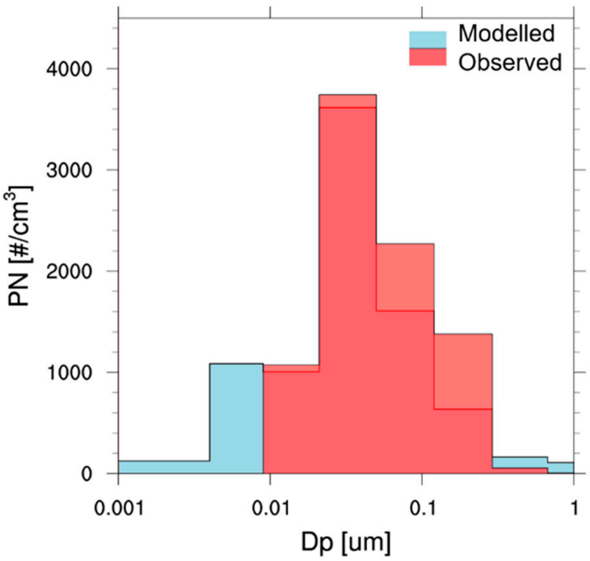

Figure S5. Comparison of the monthly mean (July 2020) size-resolved particle number concentrations (dN per size class in cm^{-3}) at aerosol supersite Marseille Longchamps (LCP) from model output of EPISODE-CityChem (light blue shaded bars) and measurements with SMPS (light red shaded bars). The x-axis presents dry particle diameter (in μm) on a logarithmic scale.

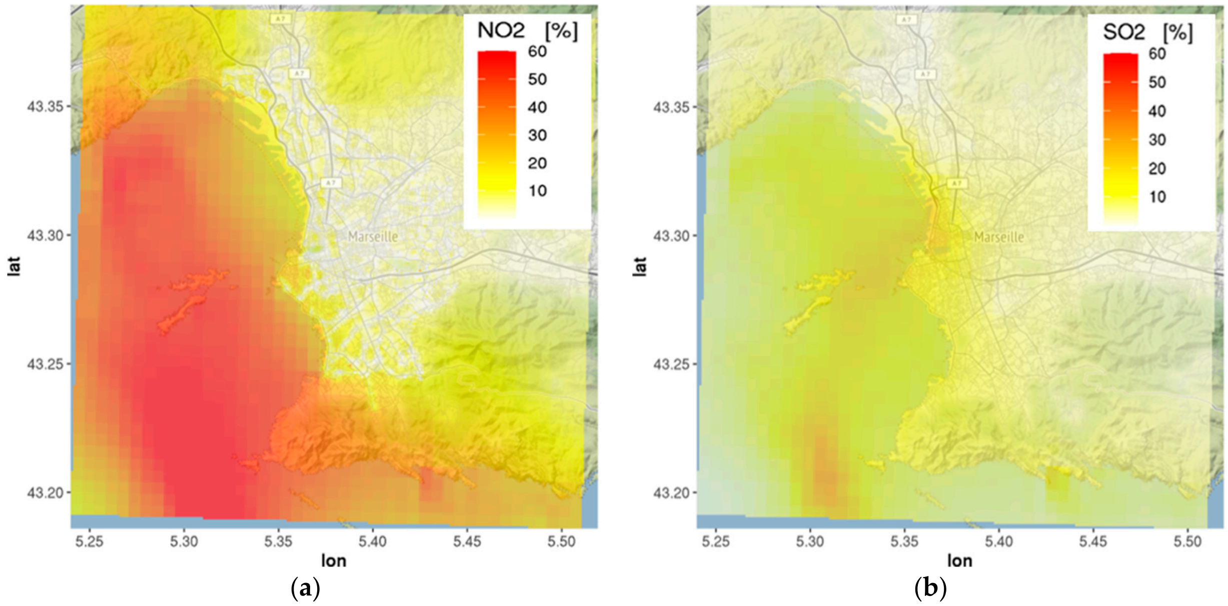

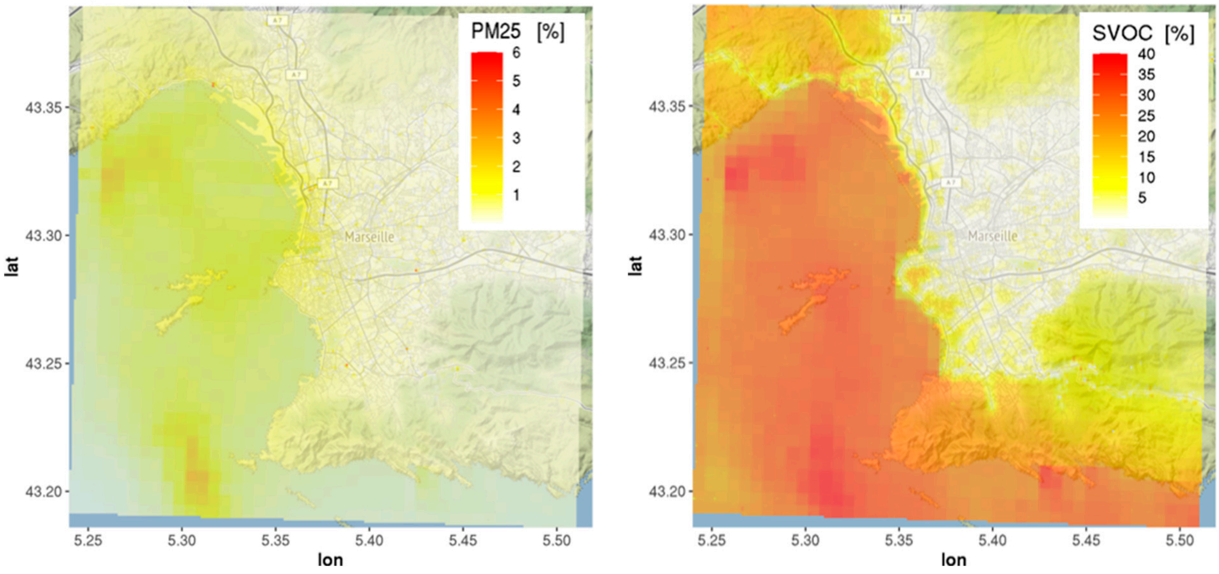

(**c**) (**d**)

Figure S6. Maps of the relative potential ship impact in Marseille (monthly mean, July 2020): (**a**) potential ship impact (%) on NO_2; (**b**) potential ship impact (%) on SO_2; (**c**) potential ship impact (%) on $PM_{2.5}$; and (**d**) potential ship impact (%) on SVOC.

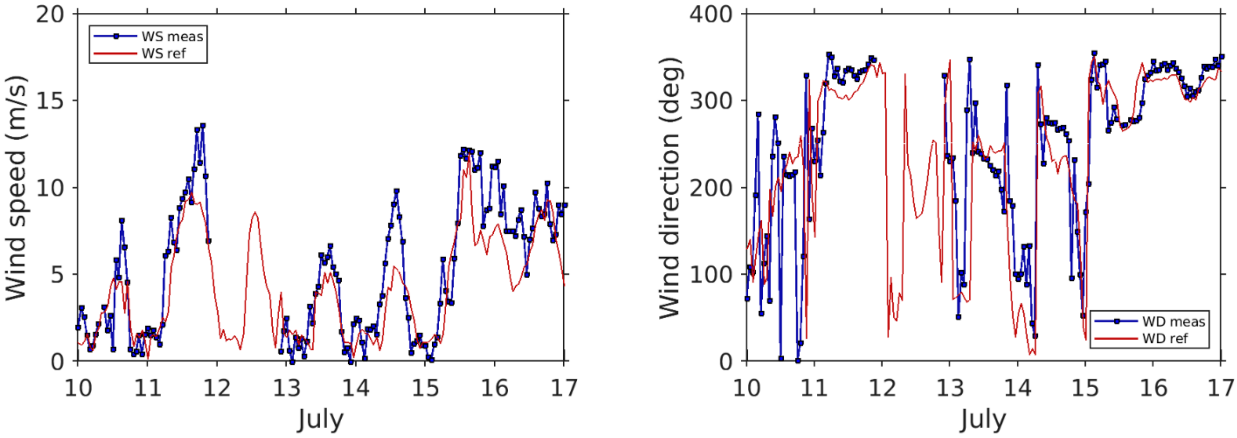

(**a**) (**b**)

Figure S7. Comparison of predicted and measured wind data at met station VIS in the period 10-17 July 2020: (**a**) wind speed (m s^{-1}); (**b**) wind direction (°). Prediction is marked as red line and measurements are marked as blue line with squares.

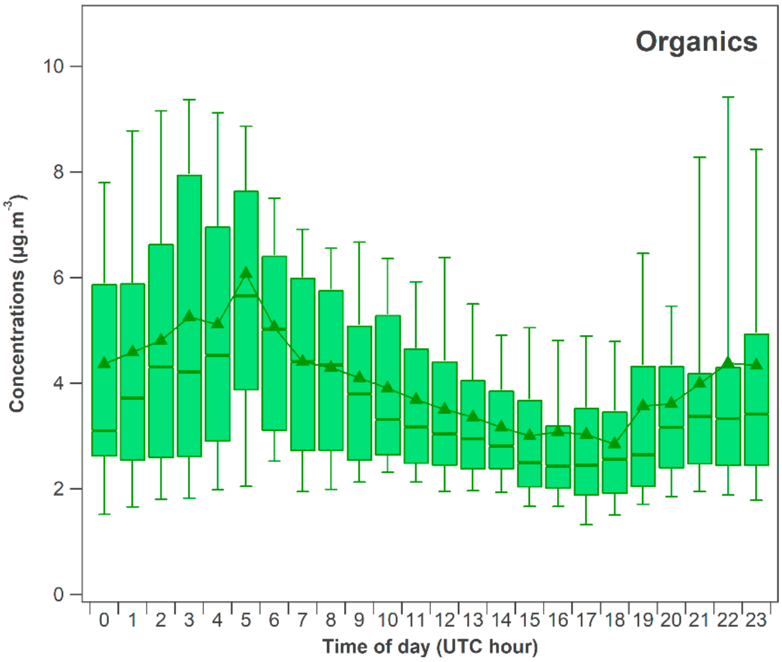

Figure S8. Diurnal variation (diel cycle) of organic matter in PM_1 measured with AMS on average of the field campaign at La Major in the period 03 to 20 July 2020. Measurement data of each hour is shown as boxes with whiskers (lower end of the box: 1st quartile, median (−), upper end of the box: 3rd quartile). Whiskers extend from the top and bottom of the box to the largest and smallest data elements, respectively. Triangles connected with a line indicate the mean value of each hour of the day.

Table S1. Particle number (PN) parameterization scheme P8P+2 in EPISODE-CityChem v1.7.

PN size class	Lower diameter (nm)	Upper diameter (nm)	Mean diameter (nm)	Bandwidth (–)	Dry dep. velocity (cm s^{-1})	Coagulation coefficient (cm^3 s^{-1})
**PN1**	1.5	4.0	2.0	1.3	1.000	6.30x10^{-9}
**PN2**	4.0	9.0	7.0	1.3	0.528	4.51x10^{-9}
**PN3**	9.0	21	17	1.5	0.355	9.76x10^{-9}
**PN4**	21	50	41	1.6	0.181	15.0x10^{-9}
**PN5**	50	120	98	1.6	0.068	5.40x10^{-9}
**PN6**	120	290	232	1.8	0.039	6.26x10^{-9}
**PN7**	290	670	551	1.8	0.031	4.27x10^{-9}
**PN8**	670	1000	850	1.8	0.023	2.28x10^{-9}
**PN9**	1000	2500	1500	2.0	0.110	0.87x10^{-9}
**PN10**	2500	10000	4000	2.0	0.200	0.80x10^{-9}

Table S2. SOA model components and their estimated saturation mass concentration and enthalpy of vaporization used in EPISODE-CityChem v1.7. Saturation concentration C^0 refers to a vapor over a pure, sub-cooled liquid at reference temperature.

Surrogate name	Description	C^0 (µg m^{-3})	ΔH_{vap} (kJ mol^{-1})
ASOA	Semi-volatile anthropogenic SOA from oxidation of XYL, C_3H_6, and nC_4H_{10}	0.0100	77.0
ALOA	Low-volatile anthropogenic SOA from oxidation of XYL, C_3H_6, and nC_4H_{10}	0.0001	77.0

BSOA	Semi-volatile biogenic SOA from oxidation of isoprene, APIN, and LIM	0.2100	138.0
BLOA	Low-volatile biogenic SOA from oxidation of isoprene, APIN, and LIM	0.0030	138.0
PIOA	Intermediate volatility delayed primary organic matter	0.1000	108.0
PSOA	Semi-volatile delayed primary organic matter	0.0006	135.0

Table S3. Split of the four VOC groups of the STEAM model to the VOC species (surrogates) of the EPISODE-CityChem model v1.7 given as mass fractions in the emissions.

STEAM VOC group	PIOC	PSOC	XYL	MEK	HCHO	CH_3CHO	C4H10	C3H6	C2
VOC A	0.914	0	0	0.011	0	0.021	0	0	0
VOC B	0	0.010	0.074	0	0.473	0.281	0.012	0.050	0.100
VOC C	0.433	0.421	0.146	0	0	0	0	0	0
VOC D	0	0.831	0.169	0	0	0	0	0	0

Table S4. Monitoring stations of the Air Quality Monitoring Network of Marseille and measured air pollutants.

Station name	Abbrev.	Station type	Coordinates	Measured air pollutants
Marseille Longchamps	LCP	urban background	43°18′18.94″ N; 5°23′41.32″ E	NO_2, NO, O_3, $PM_{2.5}$, PM_{10}, SO_2, BC
Marseille Place Verneuil	VER	traffic	43°18′32.20″ N; 5°22′04.66″ E	NO_2, NO, $PM_{2.5}$, PM_{10}, SO_2
Marseille Rabatau	RAB	traffic	43°16′32.16″ N; 5°23′47.04″ E	NO_2, NO, $PM_{2.5}$, PM_{10}
Marseille Saint Louis	STL	urban background	43°20′53.29″ N; 5°21′36.46″ E	NO_2, NO, $PM_{2.5}$, PM_{10}
Marseille Jean Moulin	JMN	traffic	43°17′16.03″ N; 5°24′02.04″ E	NO_2, NO, PM_{10}
Marseille L2 Kaddouz	KAD	suburban background	43°18′30.46″ N; 5°25′31.15″ E	NO_2, NO, PM_{10}, BC
Vallee de l'Huveaune	HUV	industrial	43°16′59.23″ N; 5°30′41.60″ E	Benzene, O_3
Aubagne Les Passons	AUB	suburban background	43°17′35.88″ N; 5°34′28.05″ E	NO_2, NO, O_3

Table S5. Main organic molecules detected by PTR-ToF-MS during the field campaign at La Major, in the port of Marseille. The corresponding model surrogate in EmChem09-HET is given if it exists.

Exact mass (m/z)	Chemical formula	Assigned chemical compound [1]	Corresponding model surrogate [2]
33.033	CH_4OH^+	Methanol	—
43.017	$C_2H_2OH^+$	Acetic acid fragment	—
43.054	$C_3H_6H^+$	Propene and HC fragments	C3H6 [3]
45.033	C_2H_4OH+	Acetaldehyde	CH_3CHO
47.049	$C_2H_6OH^+$	Ethanol	—
57.070	$C_4H_8H^+$	Butene	C3H6 [3]

59.049	$C_3H_6OH^+$	Acetone	—
61.028	$C_2H_4O_2H^+$	Acetic acid	—
69.071	$C_5H_8H^+$	Isoprene	Isoprene
71.085	$C_5H_{10}H^+$	Pentene	C3H6 [3]
73.065	$C_4H_8OH^+$	Butanone or butanal	MEK [4]
75.044	$C_3H_6O_2H^+$	Methyl acetate	—
79.054	$C_6H_6H^+$	Benzene	—
85.011	$C_6H_{12}H^+$	Hexene	C3H6 [3]
89.059	$C_4H_8O_2H^+$	Ethyl acetate	—
93.069	$C_7H_7H^+$	Toluene	XYL
101.059	$C_5H_8O_2H^+$	Pentadione	—
107.085	$C_8H_{10}H^+$	C8 Aromatics	PIOC
121.101	$C_9H_{12}H^+$	C9 Aromatics	PIOC
129.070	$C_{10}H_{18}H^+$	Naphthalene	PIOC

[1] HC: unspecified hydrocarbon.

[2] A corresponding model surrogate compound is only given if a corresponding compound exists in the chemical reaction scheme EmChem09-HET of EPISODE-CityChem.

[3] C3H6: model surrogate for C3 alkenes and higher alkenes

[4] MEK: methyl ethyl ketone, $CH_3COC_2H_5$.
